# Comparative efficacy and safety of multimodality treatment for advanced hepatocellular carcinoma with portal vein tumor thrombus: patient-level network meta-analysis

**DOI:** 10.3389/fonc.2024.1344798

**Published:** 2024-02-16

**Authors:** John Hang Leung, Shyh-Yau Wang, Henry W. C. Leung, Agnes L. F. Chan

**Affiliations:** ^1^ Department of Obstetrics and Gynecology, Ditmanson Medical Foundation Chia-Yi Christian Hospital, Chiayi, Taiwan; ^2^ Department of Radiology, An-Nan Hospital, China Medical University, Tainan, Taiwan; ^3^ Department of Radiation Oncology, An-Nan Hospital, China Medical University, Tainan, Taiwan; ^4^ Department of Pharmacy, An-Nan Hospital, China Medical University, Tainan, Taiwan

**Keywords:** patient-level NMA, multimodality treatments, hepatic arterial infusion chemotherapy, advanced or metastatic hepatocellular carcinoma, portal vein tumor thrombosis

## Abstract

**Background:**

Portal vein tumor thrombus (PVTT) is a common complication and an obstacle to treatment, with a high recurrence rate and poor prognosis. There is still no global consensus or standard guidelines on the management of hepatocellular carcinoma (HCC) with PVTT. Increasing evidence suggests that more aggressive treatment modalities, including transarterial chemoembolization, radiotherapy, targeted therapy, and various combination therapies, may improve the prognosis and prolong the survival of advanced hepatocellular carcinoma (aHCC) patients with PVTT. We aim to comprehensively review and compare the efficacy and safety of these advanced options for aHCC with PVTT.

**Methods:**

A comprehensive literature search was conducted on PubMed and EMBASE for phase II or III randomized controlled trials (RCTs) investigating multimodality treatments for aHCC with PVTT. Kaplan–Meier curves for overall survival (OS) and progression-free survival were constructed to retrieve individual patient-level data to strengthen the comparison of the benefits of all multimodality treatments of interest. Each study was pooled in a fixed-effects network meta-analysis (NMA). We also conducted subgroup analyses using risk ratios extracted from each study, including viral etiology, Barcelona Clinic Liver Cancer (BCLC) staging, alpha-fetoprotein (AFP) levels, macrovascular invasion or portal vein tumor thrombosis, and extrahepatic spread. Multimodality treatments were ranked using SUCRA scores.

**Results:**

We identified 15 randomized controlled trials with 16 multimodality regimens that met the inclusion criteria. Among them, 5,236 patients with OS results and 5,160 patients with PFS results were included in the analysis. The hepatic arterial infusion chemotherapy of fluorouracil, leucovorin, and oxaliplatin (HAIC-FO) showed OS and PFS benefits over all the other therapies. In terms of OS, HAIC-FO, nivolumab, and TACE+Len were superior to sorafenib, lenvatinib, and donatinib monotherapies, as well as HAIC-FO+Sor. In terms of PFS, TACE+Len showed better benefits than lenvatinib, donatinib, and tremelimumab+durvalumab. A low heterogeneity (*I* ^2^ < 50%) and consistency were observed. The SUCRA score for OS ranked HAIC-FO+sorafenib as the best treatment option among all multimodality treatments in hepatitis B, MVI, or PVTT with EHS and AFP 400 μg/L subgroups.

**Conclusion:**

HAIC-FO and HAIC-FO+sorafenib are statistically better options for unresectable hepatocellular carcinoma with PVTT among the multimodality treatments, and their effective and safe implementation may provide the best outcomes for HCC-PVTT patients.

## Introduction

Hepatocellular carcinoma (HCC) is one of the most common types of primary liver cancer and the third leading cause of cancer mortality, with an estimated 830,180 deaths worldwide in 2020. In Taiwan, liver and intrahepatic cholangiocarcinoma and hepatocellular carcinoma (HCC) were ranked as the second leading causes of cancer death in 2020 (1). The symptoms of early HCC are often imperceptible, and approximately 70%–80% of patients are diagnosed at an advanced stage (2). In HCC involving the invasion of intrahepatic blood vessels (portal or hepatic vein branches), patients are less tolerant to treatment and only survive for approximately 2–4 months without treatment (3, 4). Studies have found that HCC prognosis is related to the presence and extent of portal vein tumor thrombus (PVTT) (5). In addition, PVTT extension in hepatitis B virus-related HCC may involve genetic abnormalities of KDM6A, CUL9, FDG6, AKAP3, RNF139, etc. (6) Therefore, PVTT is an independent risk factor associated with a disappointing prognosis in HCC patients.

According to the Barcelona liver cancer staging system (BCLC staging), HCC with PVTT is classified as BCLC C stage. The only treatment that patients can benefit from is oral sorafenib; however, in China, Europe, and the United States, the median survival time is only 3 to 6 months and approximately 6 to 9 months (7–9). However, the BCLC staging system does not clarify the extent of PVTT, which is significantly associated with prognosis after treatment. Currently, there are only two detailed classification systems recognized globally, namely, the Chinese Cheng’s classification and the Japanese Liver Cancer Study Group’s VP staging classification system, which divide PVTT into several subgroups (10). Depending on the degree of PVTT, patients may select surgical resection, which may provide a better survival benefit. However, numerous studies have demonstrated that postoperative 5-year survival ranges from 10% to 59% (11–13). Approximately 44%–62% of patients with HCC will develop PVTT, and only a few of them will undergo a curative operation after being carefully selected. Thus, in many cases, non-operative treatment is the only available treatment option in actual clinical practice; non-operative treatment includes transarterial chemoembolization (TACE), transarterial radioembolization (TARE), hepatic arterial infusion chemotherapy (HAIC), sorafenib therapy, and radiotherapy (RT). Combinations of these treatments have also been used to improve outcomes.

Recently, HAIC combined with systemic therapies, such as sorafenib, lenvatinib, and programmed cell death protein-1 (PD-1), has been used in advanced hepatocellular carcinoma (aHCC) in phase II and III randomized controlled trials and clinical trials, and the results have shown its superiority to sorafenib monotherapy (14–19). HAIC is now accepted as a treatment option for unresectable HCC and is promoted in the clinical setting (20–22). In addition, HAIC and lenvatinib may also induce significant local immune modulation in the intrahepatic tumor microenvironment and increase the infiltration of T lymphocytes in the immunosuppressive microenvironment of HCC (23). Therefore, HAIC has been combined with several therapeutic agents and modalities, including multikinase inhibitors, immunotherapy to augment its treatment efficacy. Therefore, we performed a network meta-analysis (NMA) to comprehensively review and compare the efficacy and toxicity of these newer multimodal treatments for advanced hepatocellular carcinoma and PVTT subgroups.

## Materials and methods

### Search strategy

We performed a systematic literature search of PubMed and EMBASE databases for phase II or phase III randomized controlled trials (RCTs) investigating any targeted therapy, immunotherapy, and HAIC used alone or in combination with systemic or local therapy for the treatment of aHCC patients who had no prior history of systemic therapy. The detailed search strategies are described in the **Supplementary Materials** (online only). From the selected articles, we manually searched for additional references to identify potentially overlooked studies. This study was conducted in accordance with the Preferred Reporting Items for Systematic Reviews and Meta-Analyses (PRISMA) guidelines (24).

### Selection criteria

All trials had to meet the following criteria for inclusion: trials had to 1) be phase II or III RCTs comparing monotherapies or combination therapies of atezolizumab–bevacizumab, nivolumab, sorafenib, lenvatinib, tremelimumab+durvalumab, durvalumab, cabozantinib+atezolizumzb, donatinib, sintilimab+bevacizumab biosimilar (BevBiol), HAIC-FOLFOX (FO), HAIC plus sorafenib (HAICSor), HAIC-FO+sorafenib, TACE+lenvatinib (Len), or TACE+radiotherapy (RT), published in English from 1 January 2018 to 31 June 2022; 2) include patients with pathologically proven advanced inoperable HCC with PVTT, Barcelona Clinic Liver Cancer (BCLC) stage B or C; 3) have detailed data on methods, the characteristics of the patient population, overall survival, progression-free survival, and the disease control rate; 4) compare at least two arms that consisted of the abovementioned regimens of interest; 5) define and evaluate the disease control rate based on a comparison of abdominal computed tomography or magnetic resonance imaging before and after treatment according to the modified Response Evaluation Criteria in Solid Tumors (mRECIST) guidelines for HCC (25); and 6) present the results of severe adverse events (SAEs), defined as ≥grade 3 adverse events. Among the several publications from the same trial, only the latest or complete publication data were selected. Case reports, case series, and reviews were excluded.

### Data extraction

All eligible studies were reviewed and screened by two independent reviewers based on the study selection criteria described above. Any discrepancy was adjudicated by a third reviewer. Data from all eligible studies were extracted and summarized in a standardized table, including the study’s first author; characteristics of the population; intervention; vascular invasion; and outcomes in terms of PFS, overall survival (OS), and SAEs. In addition, we extracted primary outcome data from a subgroup of aHCC patients after we manually screened and appraised studies that did not only include PVTT aHCC patients.

### Quality assessment of the literature

The quality of the included studies was assessed by two independent investigators using the GRADE method of the Cochrane Collaboration’s tools for assessing the risk of bias, which comprises six domains: selection bias, performance bias, detection bias, attrition bias, reporting bias, and other biases. Each domain was explicitly assessed as having a low risk of bias, a high risk of bias, or an unclear risk of bias (defined as a lack of information or bias uncertainty). The risk of publication bias presented in the form of a funnel plot assesses bias in terms of the overall clinical efficacy rate using Review Manager (RevMan) software version 5.4.1 (26).

### Extraction of individual patient data

Due to the rapid advancement and novel modalities recently used in the field of aHCC treatment and the complexity of HCC etiology, more precise statistical methods are needed to make comparisons between different modalities. Therefore, a graphical reconstructive algorithm outlined by Guyot et al. (27, 28) was employed to obtain the PFS and OS data of individual patients (IPD) in each trial arm by using WebPlotDigitizer to digitize the Kaplan–Meier curves from the included studies. An IPD network meta-analysis is recognized as the gold standard approach for evidence synthesis (29, 30).

### Data analysis

This NMA included a comparison of direct and indirect treatments, and it comprehensively compared the efficacy and safety of 15 novel molecularly targeted drugs (MTDs), immunotherapy, HAIC, and their combination in the treatment of aHCC with PVTT (10–17, 31–37). All analyses were performed using the Bayesian Markov Chain Monte Carlo method in WinBUGS 1.4.3 (MRC Biostatistics Unit, Cambridge, and Imperial College School of Medicine, London, UK) and NetMetaXL (version 1.6.1) (38).

### Network meta-analysis

A network map of 16 multimodality regimens is shown in **Supplementary Figure 1**. Each node represents a regimen, and the node size is proportional to the sample size. The connecting lines represent comparisons between regimens, and the thickness of the lines is proportional to the number of studies compared. The main primary outcomes were OS, PFS, and SAEs. Bayesian Markov Chain Monte Carlo methods were used to make indirect comparisons of OS, PFS, and SAEs between the modalities of interest, and the results are presented in league tables. The relative risk of SAEs between the different regimens is presented as the odds ratio (OR) and corresponding 95% CI, with an OR >1 indicating a higher risk of SAEs than the other regimens. Cochran’s Q statistic from the fixed-effect NMA model can be decomposed into within-design heterogeneity (Q^het^) and between-design heterogeneity, which is termed design inconsistency (Qinc). DIC statistics comparison in fitted consistency and inconsistency models can be used to assess between-design heterogeneity (39). The *I^2^* test was used to assess within-study heterogeneity, with values of <50%, 50%–75%, and >75% being considered low, moderate, and considerable, respectively (40, 41).

We also conducted subgroup analyses to assess differences in OS according to HCC etiology (HBV) and the presence of PVTT or macrovascular invasion (MVI) and/or extrahepatic spread (EHS). RCTs without subgroup data were excluded, such as the Scoop-2 trial performed by Kondo et al.

Multimodality treatments were ranked according to their probability of being the best treatment based on the SUCRA score, which was calculated using the formula described in Salanti, Ades, and Ioannidis (2011) (42). The SUCRA values were obtained from the distribution of ranking probabilities. The higher the SUCRA value and close to 100%, the higher the treatment ranking in the network.

### Sensitivity analysis

We re-ran the model to compare the results by including and excluding one study with a potential high risk of bias, with forest plots describing the effect estimates for the paired multimodality regimens in the sensitivity analysis.

## Results

### Characteristics of the included RCTs

Fifteen RCTs containing 16 modality regimens with a total of 5,638 patients and 5,562 patients with OS and PFS results were identified through searches of the PubMed and EMBASE electronic databases (online **Supplementary Figure 2**). The study and patient characteristics are presented in **Table 1** (14–17, 31–37, 43–47).

**Table 1:** Baseline characteristics of the population included in the clinical trials of interests.

	Study	Treatment (*n*)	Study design	Median age	Etiology HBV/HCV	BCLC stage B/C, *N*	PVTT, *N* (%)	Extrahepatic metastasis, *N* (%)	Serum AFP ≧400 g/mL (*N*)	Primary outcomes	Tx cycles median	Treatment duration (weeks)
	PD-1 inhibitor+anti-VEGF vs. sorafenib											
**1**	**Cheng 2022** (**43**) **※**	Ate+Bev = 336 Sorafenib = 165	Phase III	64 (56–71) 66 (59–71)	129/48; 62/26	B/C: 52/276 B/C: 26/133	129 (38) 71 (43)	212 (63) 93 (56)	126 61	PFS, OS, SAE	Ate: 5 Bev 4	6
	HAIC vs. sorafenib											
**2**	**Lyu, 2022** (**32**) **FOHAIC-1**※	HAIC = 130 Sorafenib = 132	Phase III	54 (45–61) 53 (45–62)	120/2 114/4	B/C: 5/125 B/C: 9/123	82 (55.8) 80 (54.4)	75 (51) 80 (54.4)	69 64	PFS, OS, SAE	HAIC: 8	10.4 (HAIC) 14 (sorafenib)
**3**	**Choi, 2018** (**31**) **※**	HAIC = 29 Sorafenib = 29	Randomized prospective	60.3 ± 9.5 60.2 ± 7.3	21/0 8/5	NA	29 (100) 29 (100)	NA	29 29	PFS, OS, SAE	HAIC 4.4	17.6
	Sorafenib+HAIC (SoraHAIC) vs. sorafenib											
**4**	**Kudo, 2018** (**14**) **(SILIUS)**※	SoraHAIC = 102 Sorafenib = 103	Phase III	69 (62–75) 68 (62–75)	26/47 22/76	B/C: 32/27 B/C: 70/76	64 (62) 58 (57)	26 (25) 27 (27)	42 35	PFS, OS, SAE	2	19.2
**5**	**He MK, 2019** (**15**) **※**	SoraHAIC = 125 Sorafenib = 122	Randomized clinical trial	49 (41–55) 49 (40–56)	62/101 89/99	NA	125 (100) 122(100)	38/125 (30.4) 42/122 (34.4)	**+**	PFS, OS, SAE	4	19.6 (SoraHAIC) 8.14 (sorafenib)
**6**	**Kondo, 2019** (**16**) **Scoop-2**	SoraHAIC = 35 Sorafenib = 33	Phase II	72.0 ± 7.0 70.9 ± 9.1	4/20 3/21	B/C: 14/19 B/C: 13/18	21 (60) 22 (67)	10 (29) 8 (24)	**+**	PFS, OS, SAE	2.2	11.2
**7**	**Zheng, 2022** (**17**) **※**	SoraHAIC = 32 Sorafenib = 32	Phase II	56 ± 11 55 ± 10	28/2 29/3	NA	32 (100) 32 (100)	4 (12) 5 (16)	+	PFS, OS, SAE	5.5	28
	Lenvatinib vs. sorafenib											
**8**	**Kudo, 2018** (**44**) **※(REFLECT)**	Lenvatinib = 478 Sorafenib = 476	Phase III	63 (20–88) 62 (22–88)	251/91: 53/19 228/126: 21/45	B/C: 104/374 B/C: 92/384	109 (23%) 90 (19%)	291 (61) 295 (62)	AFP ≧200 183/154	PFS, OS, SAE	2	8
	Nivolumab vs. sorafenib											
**9**	**Yau, 2022** (**45**) **※** **(CheckMate 459)**	Nivolumab = 371 Sorafenib = 372	Phase III	65(57–71) 65 (58–72)	116/87 117/86	B/C: 53/303 B/C: 63/291	124 (33) 118 (32)	222 (60) 207 (56)	124 142	PFS, OS, SAE	3.5	7
	Donatinib vs. sorafenib											
**10**	**Qin, 2021** (**33**)	Donatinib = 328 Sorafenib = 331	Phase III	53 (46–62) 53 (46–63)	293/7 301/5	B/C: 42/286 B/C: 41/290	241 (73) 243 (73)	241 (73) 243 (73)	173 177	PFS, OS, SAE	7.4	14.8
	Cabozantinib+Ate vs. sorafenib											
**11**	**Kelley, 2022 (** 35) **(COSMIC-32)**	Cabozantinib+Ate = 432 Sorafenib = 217 Cabozantinib = 188	Phase III	64 (58–70) 64 (57–71) 64 (58–71)	127/136 64/67 59/60	B/C: 140/292 B/C: 72/145 B/C: 66/122	84 (19) 35 (16) 40 (21)	232 (54) 122 (56) 102 (54)	163 65 65	PFS, OS, SAE	8	24
	Tremelimumab+durvalumab vs. durvalumab or sorafenib											
**12**	**Abou-Alfa, 2022** (**37**)	Tremelimumab+durvalumab = 393 vs. durvalumab = 389 or sorafenib = 389	Phase III	65 (22–86) 64 (20–86) 64 (18–88)	122/110 119/107 119/104	B/C: 77/316 B/C: 80/309 B/C: 66/323	103 (26.2) 94 (24.2) 100 (25.7)	209 (53.2) 212 (54.5) 203 (52.2)	145 (16.9) 137 (35.2) 124 (31.9)	PFS, OS, SAE	5	19.6
	Sintilimab plus Bev biosimilar vs. sorafenib											
**13**	**Ren, 2021** (**34**)	Sintilimab plus BevBiol vs. sorafenib	Phase III	53 (21–82) 54 (28–77)	359/6 179/8	B/C: 56/324 B/C: 27/164	105 (80) 50 (26)	279 (73) 144 (75)	165 (43) 81 (42)	PFS, OS, SAE	7 4	22 vs. 12.4
	TACE+radiotherapy (RT) vs. sorafenib											
**14**	**Yoon, 2018** (**36**)	TACE+RT Sorafenib	Phase III	55 (42–77) 55(33–82)	36/1 40/0	B/C: NA	45 (100) 45 (100)	8 (17.8) 0 (0)	+	PFS, OS, SAE	5 2	31 (TACE) 11.7 (RT)
**15**	**TACE+lenvatinib vs. Lenvatinib**											
	**Peng, 2023** (**46**)	TACE+lenvatinib	Phase III	56 (48–63)	148/4	NA	122 (72)	94 (55)	83 (49)	PFS, OS	4	32.8
		Lenvatinib		54 (46.0–64.)	144/6		117 (70)	95 (57)	87 (52)	SAE	5.1	20.4
**16**	**Ding, 2021** (**47**)	TACE+lenvatinib TACE+sorafenib	Phase III	57 ± 11 56 ± 11	30/1 29/3	NA	32 (100) 32 (100)	13 (91) 9 (28)	16 (50) 18 (56)	PFS, OS, SAE	6.9 3.0	18.8 12.4

N, number; BCLC, Barcelona Clinic Liver Cancer; HBV/HCV, Hepatitis B virus (B)/ Hepatitis C virus (C); AFP, alpha fetoprotein; PVTT, portal vein tumor thrombosis; OS, overall survival; PFS, progression free survival; SAE, severe adverse event; PVTT, Portal vein tumor thrombosis; FOLFOX, Oxaliplatin+Leucovorin+5-FU); ※, The study focused on patients with AHCC with PVTT or showed subgroup analysis data. HAIC, Hepatic arterial infusion chemotherapy; Tx, treatment.

**Figure 1:**
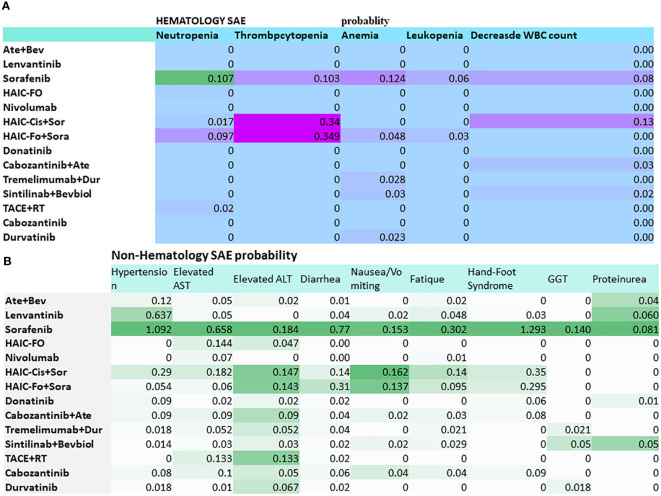
Heatmap of grade 3–5 toxicity spectra based on each of the specific adverse events for multidisciplinary treatment in advanced hepatocellular carcinoma with PVTT. **(A)** Hematology SAE. **(B)** Non-hematology SAE. Abbreviations: Ate+Bev, atezolizumab–bevacizumab; HAIC-FO, hepatic arterial infusion chemotherapy plus FOLFOX; HAIC-Cis+Sor, HAIC (cisplatin+5Fu or cisplatin) plus sorafenib; Dur, durvalumab; RT, radiotherapy. The deep color presented a higher risk.

Among all the studies, 12 studies were phase III randomized clinical trials, 2 were phase II RCTs, and 2 were RCTs. The publication years of the included studies ranged from 2018 to 2023, with updated outcome data. The median age of the patients was 49 to 72 years old. The age of the patients in the Scoop-2 phase II trial was slightly higher (72.0 ± 7.0) than in the other studies. All studies were conducted in the Asia-Pacific region, comprising 20 countries; therefore, they included patients from the Asia-Pacific, European, and North American regions. The patients from the Asia-Pacific region represented 67% in REFLECT, 50.4% in IMbrave150, and 40% in CheckMate 459. The proportion of patients with HBV ranged from 8.6% to 94%, while 1.5%–32% had HCV. In total, 5 of the 15 RCTs included patients with 100% PVTT. The percentage of patients with PVTT in the remaining RCTs ranged from 19% to 80% (**Table 1**). The probabilities of SAEs are presented in a heatmap in **Figure 1**.

### Overall survival

HAIC-FO showed a statistically significant OS benefit over all modalities of interest, except for nivolumab, TACE+Len, TACE+Sor, tremelimumab+Dur, and Ate+Bev. Three modalities were superior to sorafenib: HAIC-FO (OR = 0.28, 95% CI = 0.11–0.64), nivolumab (OR = 0.54, 95% CI = 0.36–0.79), and TACE+Len (OR = 0.78, 95% CI = 0.66–0.92) (**Table 2**). A low heterogeneity (*I* ^2 =^ 38%) and no evidence of inconsistency (each study data point must have a posterior mean deviance contribution of approximately 1) were observed, indicating consistency (**Supplementary Figures 3B**).

**Table 2:** Network comparisons of outcomes among different treatments.

PFS														
**HAIC-FO**														
0.42 (0.13 – 1.32)	**TACE+Len**													
0.41 (0.13 – 1.21)	0.97 (0.47 – 2.00)	**HAIC+Sor**												
0.49 (0.10 – 2.37)	1.16 (0.39 – 3.49)	1.21 (0.32 – 4.47)	**TACE+ Sor**											
0.39 (0.13 – 1.08)	0.91 (0.48 – 1.75)	0.94 (0.55 – 1.60)	0.78 (0.22 – 2.78)	**Donatinib**										
0.39 (0.12 – 1.21)	0.93 (0.43 – 2.04)	0.96 (0.48 – 1.95)	0.80 (0.21 – 3.08)	1.02 (0.55 – 1.91)	**Ate+Bev**									
0.31 (0.11 – 0.83)	0.73 (0.41 – 1.30)	0.76 (0.49 – 1.17)	0.63 (0.18 – 2.16)	0.81 (0.60 – 1.10)	0.79 (0.45 – 1.35)	**Sorafenib**								
0.30 (0.09 – 0.92)	0.70 (0.32 – 1.57)	0.73 (0.36 – 1.48)	0.61 (0.16 – 2.33)	0.78 (0.41 – 1.46)	0.76 (0.35 – 1.65)	0.96 (0.56 – 1.68)	**Cabozantinib+Ate**							
0.29 (0.09 – 0.83)	0.69 (0.36 – 1.36)	0.72 (0.41 – 1.24)	0.59 (0.16 – 2.13)	0.76 (0.48 – 1.19)	0.75 (0.39 – 1.41)	0.94 (0.67 – 1.32)	0.98 (0.51 – 1.87)	**Nivolumab**						
0.29 (0.09 – 0.84)	0.69 (0.33 – 1.40)	0.71 (0.38 – 1.30)	0.59 (0.16 – 2.18)	0.75 (0.44 – 1.27)	0.74 (0.37 – 1.46)	0.93 (0.61 – 1.43)	0.97 (0.48 – 1.96)	0.99 (0.57 – 1.71)	**Sintiliman+Bevbiol**					
0.29 (0.09 – 0.84)	0.67 (0.33 – 1.39)	0.70 (0.37 – 1.31)	0.58 (0.16 – 2.17)	0.74 (0.43 – 1.27)	0.73 (0.36 – 1.46)	0.92 (0.59 – 1.43)	0.96 (0.46 – 1.94)	0.97 (0.56 – 1.70)	0.99 (0.53 – 1.85)	**HAIC-FO+Sor**				
0.26 (0.07 – 0.95)	0.62 (0.22 – 1.71)	0.64 (0.25 – 1.65)	0.53 (0.12 – 2.36)	0.68 (0.27 – 1.66)	0.66 (0.24 – 1.82)	0.84 (0.36 – 1.95)	0.88 (0.31 – 2.36)	0.89 (0.36 – 2.21)	0.90 (0.35 – 2.29)	0.92 (0.35 – 2.36)	**TACE+RT**			
0.25 (0.08 – 0.70)	0.58 (0.38 – 0.90)	0.60 (0.34 – 1.07)	0.50 (0.15 – 1.62)	0.64 (0.40 – 1.03)	0.63 (0.32 – 1.20)	0.79 (0.55 – 1.14)	0.83 (0.43 – 1.58)	0.84 (0.51 – 1.38)	0.85 (0.48 – 1.49)	0.86 (0.49 – 1.53)	0.94 (0.37 – 2.38)	**Lenvatinib**		
0.21 (0.07 – 0.61)	0.49 (0.24 – 0.99)	0.51 (0.28 – 0.93)	0.42 (0.11 – 1.56)	0.54 (0.32 – 0.90)	0.53 (0.27 – 1.04)	0.67 (0.44 – 1.02)	0.70 (0.35 – 1.39)	0.71 (0.42 – 1.21)	0.72 (0.39 – 1.31)	0.73 (0.40 – 1.36)	0.80 (0.31 – 2.06)	0.85 (0.49 – 1.46)	**Durvalumab**	
0.18 (0.05 – 0.52)	0.41 (0.20 – 0.84)	0.43 (0.23 – 0.78)	0.36 (0.09 – 1.32)	0.45 (0.27 – 0.77)	0.45 (0.22 – 0.88)	0.56 (0.36 – 0.86)	0.59 (0.29 – 1.17)	0.60 (0.35 – 1.03)	0.60 (0.33 – 1.11)	0.61 (0.33 – 1.14)	0.67 (0.26 – 1.74)	0.71 (0.40 – 1.25)	0.84 (0.53 – 1.32)	**Tremelimumab+ Dur**
OS														
**HAIC-FO**														
0.51 (0.19 – 1.31)	**Nivolumab**													
0.52 (0.18 – 1.47)	1.02 (0.51 – 2.02)	**TACE+Len**												
0.60 (0.13 – 2.51)	1.16 (0.34 – 3.90)	1.14 (0.42 – 3.15)	**TACE+Sor**											
0.42 (0.16 – 1.04)	0.82 (0.50 – 1.35)	0.81 (0.41 – 1.57)	0.70 (0.21 – 2.39)	**Tremelimumab+Dur**										
0.38 (0.14 – 1.00)	0.74 (0.41 – 1.35)	0.73 (0.35 – 1.52)	0.64 (0.18 – 2.22)	0.91 (0.52 – 1.58)	**Ate+Bev**									
0.33 (0.13 – 0.81)	0.64 (0.38 – 1.05)	0.63 (0.32 – 1.23)	0.55 (0.16 – 1.87)	0.78 (0.57 – 1.06)	0.86 (0.49 – 1.48)	**Durvalumab**								
0.32 (0.12 – 0.80)	0.62 (0.35 – 1.07)	0.61 (0.30 – 1.22)	0.53 (0.15 – 1.83)	0.75 (0.46 – 1.25)	0.83 (0.45 – 1.51)	0.97 (0.58 – 1.61)	**Cabozantinib+Ate**							
0.28 (0.11 – 0.64)	0.54 (0.36 – 0.79)	0.53 (0.30 – 0.95)	0.46 (0.14 – 1.51)	0.66 (0.48 – 0.90)	0.72 (0.46 – 1.13)	0.84 (0.61 – 1.16)	0.87 (0.59 – 1.29)	**Sorafenib**						
0.25 (0.07 – 0.82)	0.49 (0.20 – 1.22)	0.49 (0.17 – 1.35)	0.42 (0.10 – 1.79)	0.60 (0.25 – 1.46)	0.66 (0.26 – 1.69)	0.77 (0.32 – 1.87)	0.80 (0.31 – 2.02)	0.91 (0.40 – 2.09)	**TACE+RT**					
0.26 (0.10 – 0.65)	0.51 (0.31 – 0.84)	0.50 (0.26 – 0.98)	0.44 (0.13 – 1.49)	0.62 (0.40 – 0.98)	0.69 (0.39 – 1.19)	0.80 (0.51 – 1.25)	0.82 (0.49 – 1.39)	0.95 (0.69 – 1.31)	1.04 (0.43 – 2.55)	**Donatinib**				
0.26 (0.09 – 0.68)	0.50 (0.28 – 0.89)	0.49 (0.24 – 1.02)	0.43 (0.13 – 1.52)	0.61 (0.36 – 1.05)	0.68 (0.36 – 1.26)	0.79 (0.46 – 1.35)	0.81 (0.45 – 1.47)	0.93 (0.61 – 1.44)	1.02 (0.40 – 2.62)	0.98 (0.58 – 1.69)	**Sintilimab+BevBiol**			
0.26 (0.10 – 0.65)	0.51 (0.30 – 0.85)	0.50 (0.32 – 0.77)	0.43 (0.14 – 1.33)	0.61 (0.38 – 1.00)	0.68 (0.38 – 1.21)	0.79 (0.48 – 1.30)	0.82 (0.48 – 1.41)	0.94 (0.65 – 1.35)	1.03 (0.42 – 2.56)	0.99 (0.61 – 1.62)	1.01 (0.58 – 1.76)	**Lenvatinib**		
0.24 (0.08 – 0.65)	0.47 (0.24 – 0.90)	0.46 (0.21 – 1.01)	0.40 (0.11 – 1.45)	0.57 (0.31 – 1.06)	0.63 (0.31 – 1.27)	0.74 (0.40 – 1.37)	0.76 (0.39 – 1.47)	0.87 (0.51 – 1.48)	0.96 (0.36 – 2.57)	0.92 (0.50 – 1.71)	0.94 (0.47 – 1.86)	0.93 (0.48 – 1.77)	**HAIC+ Sor**	
0.19 (0.07 – 0.48)	0.36 (0.20 – 0.64)	0.35 (0.17 – 0.74)	0.31 (0.09 – 1.10)	0.44 (0.25 – 0.76)	0.48 (0.25 – 0.90)	0.56 (0.33 – 0.98)	0.58 (0.32 – 1.06)	0.67 (0.42 – 1.04)	0.73 (0.28 – 1.86)	0.70 (0.40 – 1.22)	0.71 (0.38 – 1.33)	0.71 (0.40 – 1.26)	0.76 (0.38 – 1.54)	**HAIC-FO+ Sor**
SAE														
**Nivolumab**														
0.55 (0.36 – 0.85)	**Durvalumab**													
0.61 (0.21 – 1.95)	1.11 (0.37 – 3.50)	**TACE+RT**												
0.46 (0.29 – 0.72)	0.83 (0.54 – 1.27)	0.74 (0.24 – 2.22)	**Donatinib**											
0.35 (0.11 – 1.18)	0.64 (0.20 – 2.11)	0.57 (0.12 – 2.74)	0.77 (0.24 – 2.60)	**TACE+Sor**										
0.32 (0.21 – 0.49)	0.58 (0.43 – 0.77)	0.52 (0.17 – 1.54)	0.70 (0.46 – 1.07)	0.90 (0.28 – 2.87)	**Tremelimumab+Dur**									
0.30 (0.21 – 0.41)	0.54 (0.40 – 0.71)	0.48 (0.16 – 1.37)	0.65 (0.47 – 0.89)	0.84 (0.26 – 2.62)	0.93 (0.70 – 1.23)	**Sorafenib**								
0.22 (0.14 – 0.36)	0.41 (0.26 – 0.64)	0.36 (0.11 – 1.10)	0.49 (0.30 – 0.79)	0.63 (0.19 – 2.08)	0.70 (0.45 – 1.10)	0.76 (0.53 – 1.07)	**Sintilimab+BevBiol**							
0.22 (0.13 – 0.36)	0.39 (0.24 – 0.64)	0.35 (0.11 – 1.07)	0.47 (0.28 – 0.79)	0.61 (0.18 – 2.04)	0.67 (0.41 – 1.10)	0.73 (0.49 – 1.09)	0.96 (0.56 – 1.64)	**Ate+Bev**						
0.20 (0.13 – 0.30)	0.36 (0.24 – 0.53)	0.32 (0.10 – 0.95)	0.43 (0.28 – 0.65)	0.55 (0.18 – 1.67)	0.61 (0.41 – 0.91)	0.66 (0.50 – 0.88)	0.88 (0.56 – 1.37)	0.91 (0.56 – 1.49)	**Lenvatinib**					
0.16 (0.09 – 0.30)	0.29 (0.16 – 0.53)	0.26 (0.08 – 0.85)	0.35 (0.19 – 0.65)	0.46 (0.16 – 1.24)	0.51 (0.28 – 0.91)	0.54 (0.32 – 0.92)	0.72 (0.39 – 1.34)	0.75 (0.38 – 1.44)	0.82 (0.53 – 1.27)	**TACE+Len**				
0.14 (0.08 – 0.25)	0.26 (0.15 – 0.45)	0.23 (0.07 – 0.74)	0.31 (0.18 – 0.54)	0.40 (0.11 – 1.36)	0.45 (0.26 – 0.77)	0.48 (0.30 – 0.76)	0.63 (0.36 – 1.14)	0.66 (0.36 – 1.22)	0.73 (0.42 – 1.25)	0.88 (0.44 – 1.77)	**HAIC-FO+ Sor**			
0.13 (0.08 – 0.20)	0.23 (0.15 – 0.36)	0.21 (0.06 – 0.62)	0.28 (0.17 – 0.45)	0.36 (0.11 – 1.18)	0.40 (0.25 – 0.62)	0.43 (0.30 – 0.61)	0.57 (0.34 – 0.93)	0.59 (0.34 – 1.01)	0.65 (0.41 – 1.01)	0.78 (0.42 – 1.48)	0.89 (0.50 – 1.58)	**Cabozantinib+Ate**		
0.08 (0.04 – 0.15)	0.15 (0.08 – 0.27)	0.13 (0.04 – 0.43)	0.18 (0.09 – 0.33)	0.23 (0.06 – 0.81)	0.25 (0.14 – 0.47)	0.27 (0.16 – 0.47)	0.36 (0.19 – 0.69)	0.38 (0.19 – 0.74)	0.41 (0.22 – 0.76)	0.50 (0.23 – 1.07)	0.57 (0.28 – 1.17)	0.64 (0.33 – 1.23)	**HAIC-FO**	
0.04 (0.02 – 0.08)	0.08 (0.04 – 0.15)	0.07 (0.02 – 0.23)	0.10 (0.05 – 0.18)	0.12 (0.03 – 0.44)	0.14 (0.07 – 0.25)	0.15 (0.09 – 0.25)	0.20 (0.10 – 0.38)	0.21 (0.10 – 0.40)	0.23 (0.12 – 0.41)	0.27 (0.13 – 0.58)	0.31 (0.15 – 0.64)	0.35 (0.18 – 0.67)	0.55 (0.25 – 1.18)	**HAIC+ Sor**

Comparisons should be read from left to right. Cells narked in red color are significant (OR<1). For OS, PFS, and SAE an OR < 1.

HAIC, hepatic arterial infusion chemotherapy; TACE, transarterial chemoembolization; RT, external beam radiotherapy; Sor, Sorafenib; Sor, Sorafenic; Der, dervalumab; Ate, atezolizumab; FO, FOLFOX.

**Figure 2:**
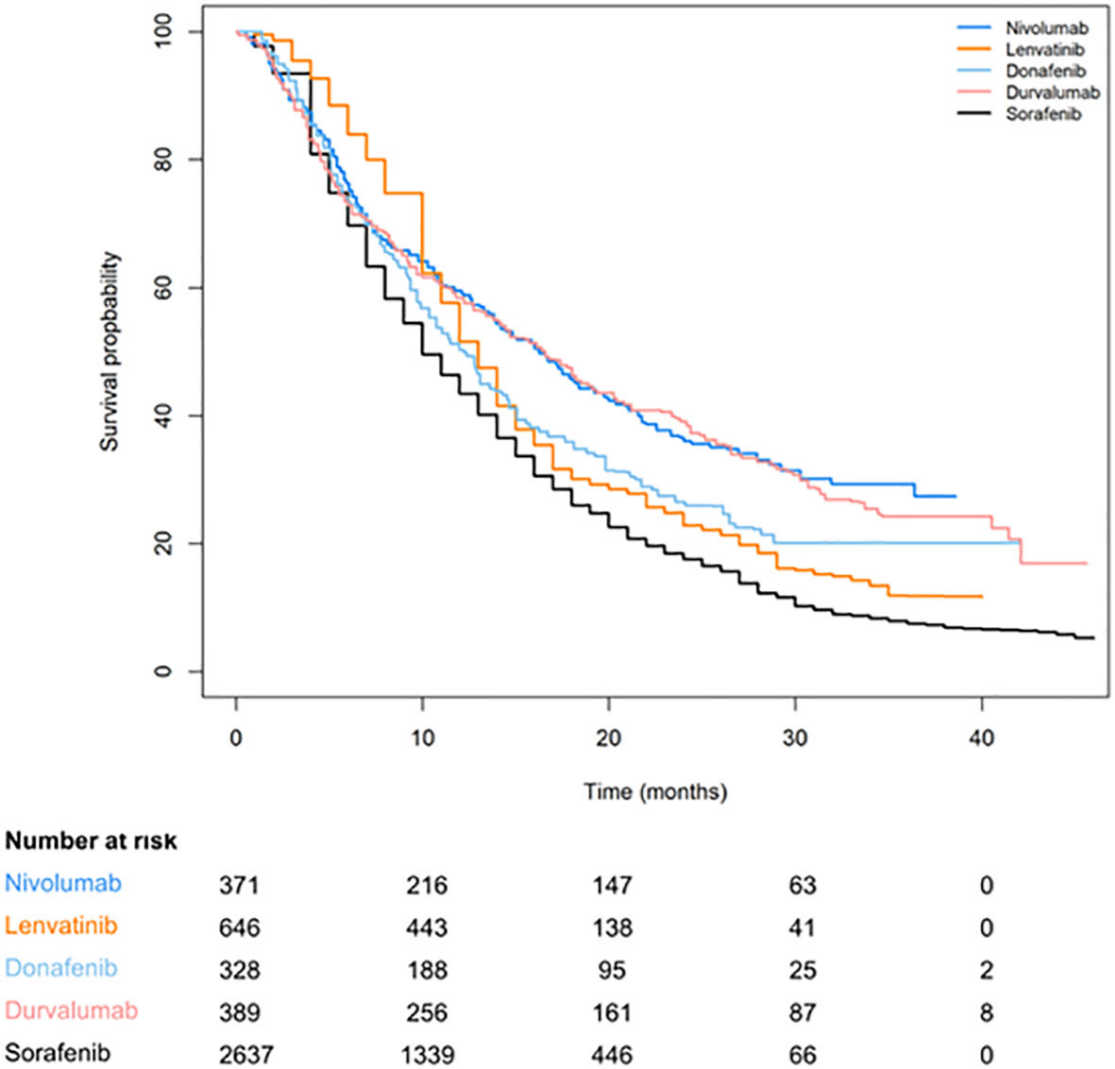
Reconstructed Kaplan–Meier curves of OS for individual patient data extracted from single-agent systemic therapies for aHCC.

**Figure 3:**
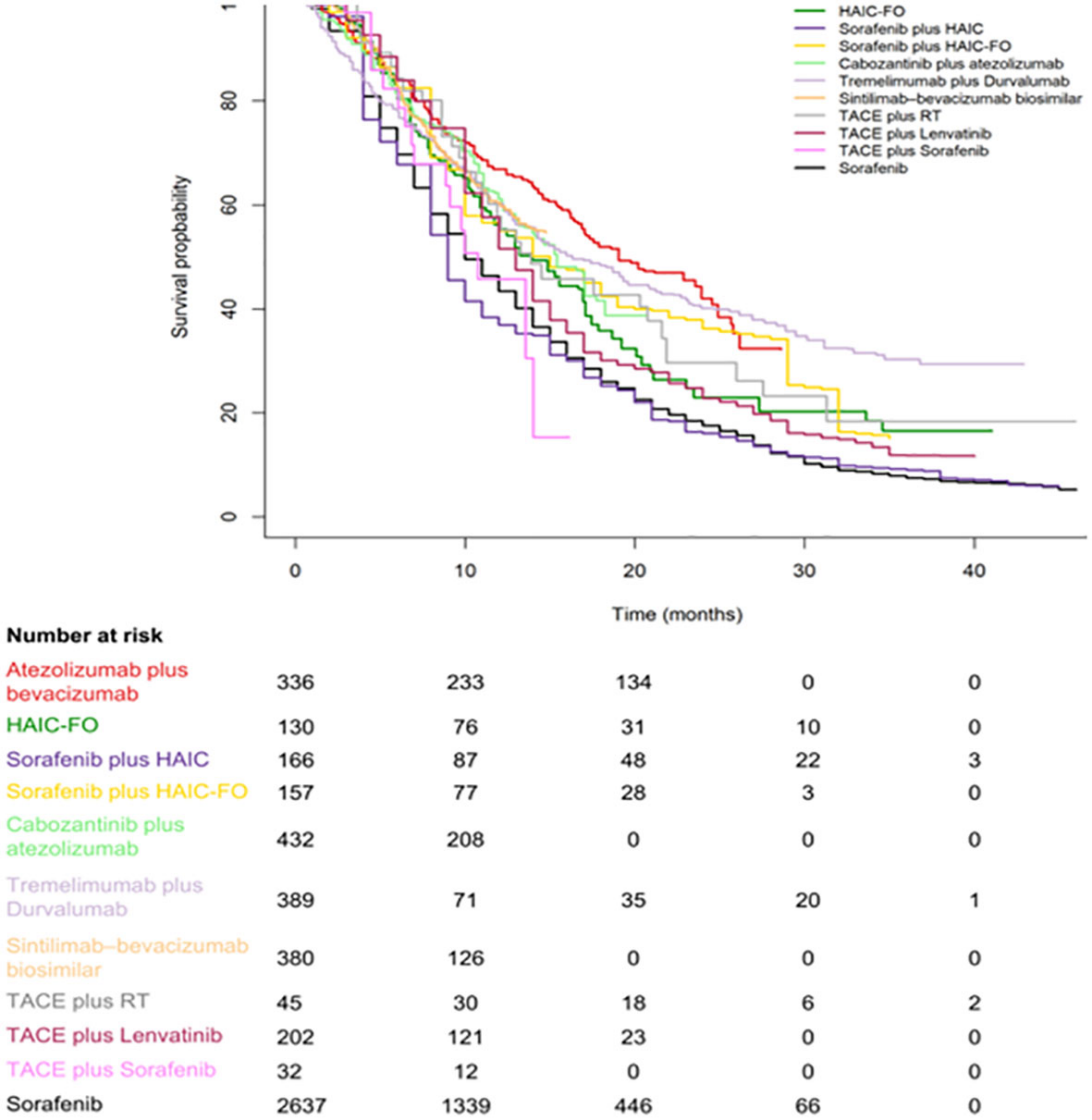
Kaplan–Meier curves of OS for individual patient data extracted from combination systemic therapies for aHCC.

**Figures 2**, **3** are pooled reconstructed Kaplan–Meier curves of the OS of monotherapies and combined transarterial therapies. A visual examination of **Figure 2** shows that durvalumab and nivolumab provide long-term benefits over sorafenib, donatinib, and lenvatinib, and tremelimumab+Dur also shows long-term benefits relative to the other combination regimens shown in **Figure 3**.

### Progression-free survival

HAIC-FO was significantly superior to all modalities of interest, except for TACE+Len (OR = 0.42, 95% CI = 0.13–1.32), HAIC+Sor (OR = 0.41, 95% CI = 0.13–1.21), and TACE+Sor (OR = 0.49, 95% CI = 0.10–2.37) (**Table 2**). TACE+Len and HAIC+Sor were significantly better than lenvatinib, durvalumab, and tremelimumab+durvalumab. In addition, donatinib was also favored over durvalumab (OR = 0.54, 95% CI = 0.32–0.90) and tremelimumab+Dur (OR = 0.45, 95% CI = 0.27–0.77). A low heterogeneity (*I* ^2^ = 9%) and no evidence of inconsistency (each study data point must have a posterior mean deviance contribution of approximately 1) were observed, indicating consistency (**Supplementary Figures 4A**). Kaplan–Meier curves of the PFS of the monotherapy regimens and combination regimens and pooled plots of the PFS and OS of all multimodality regimes are shown in **Supplementary Figures 5–8**.

Online **Supplementary Figure 9** shows the SUCRA score plot for OS versus PFS. HAIC-FO ranked higher in OS and PFS, while HAIC+sorafenib had a higher ranking in PFS (SUCRA score: 0.7893) than OS (SUCRA score: 0.3313).

### Quality assessment of the studies

**Figure 4:**
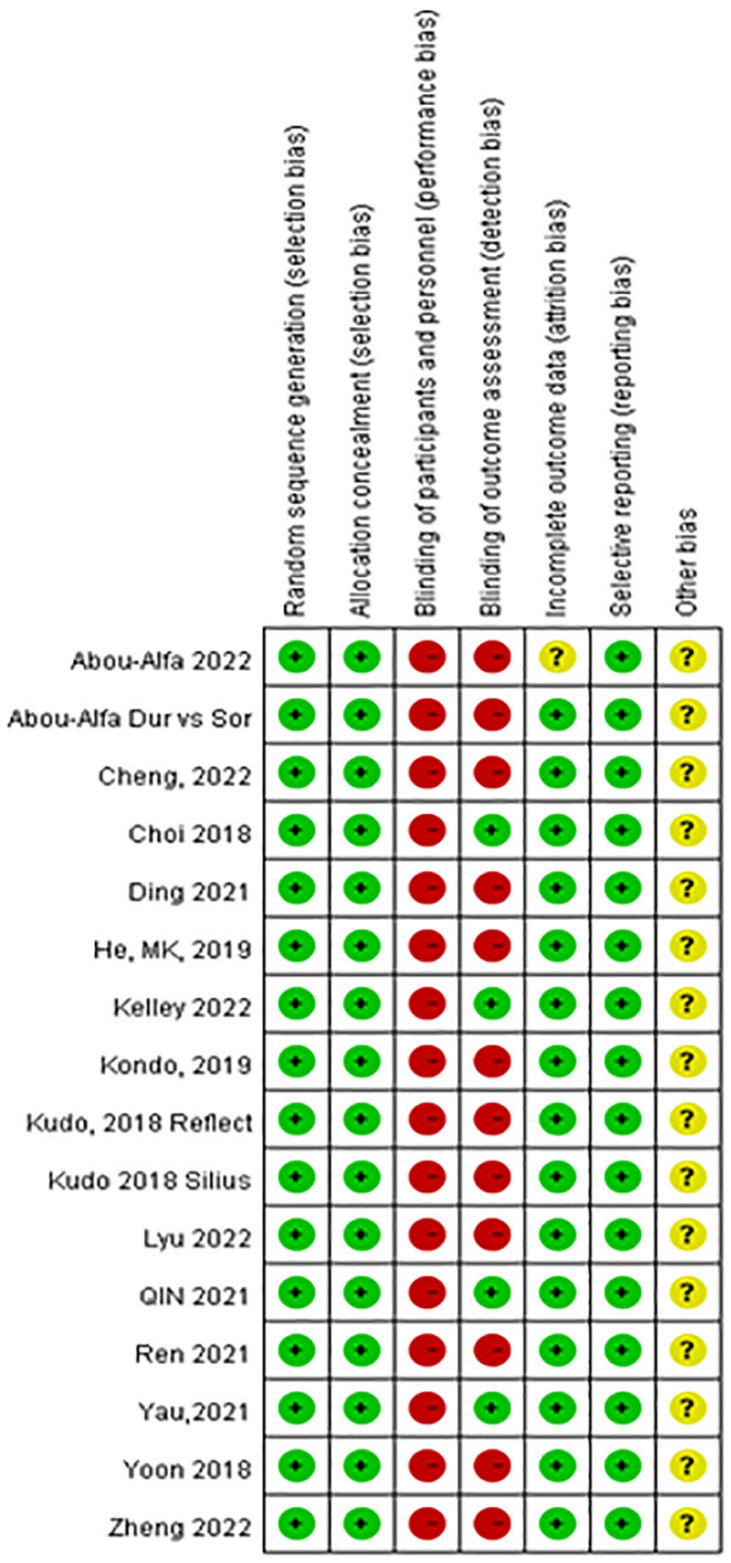
Risk of bias of the included 15 randomized control trials [review authors’ judgments about each risk of bias item for each included study: low risk (+), high risk (−), and unclear risk ()?].

**Figure 4** shows the risk of bias in the 15 RCTs included in this network meta-analysis. All included studies were determined to have high-quality evidence according to the criteria for risk of bias using the GRADE method, with all reporting random sequence generation and concealed allocation. No studies reported the blinding of participants and personnel. Four studies clearly mentioned specific methods used for the blinding of the outcome assessor. Overall, study quality was assessed as high because of the low heterogeneity determined based on I^2^ <50% (9% for PFS; 38% for OS) (**Supplementary Figure 7**), and no inconsistency was observed because all studies were along the line of equality (**Supplementary Figure 4**).

**Figure 5:**
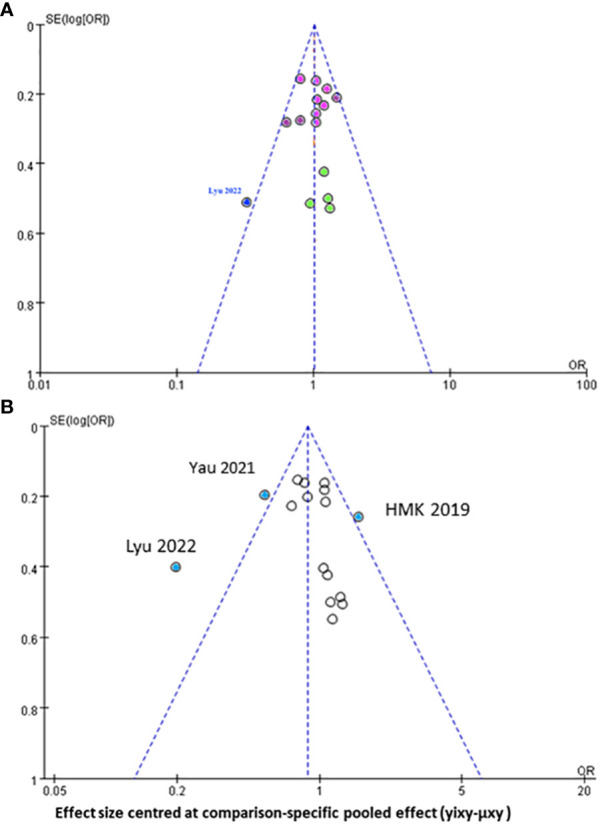
Funnel plot for the assessment of publication bias for **(A)** progression free survival **(B)** overall survival of all mutimodal regimens in network meta-analysis.

Regarding the funnel plot for the assessment of publication bias in the network meta-analysis (**Figure 5**), the central line suggests the null hypothesis that study-specific effect sizes do not differ from the respective comparison-specific pooled effect estimates. The dots in different colors represent the comparisons of different regimens. All the dots are evenly distributed on both sides of the funnel plot and symmetrical, indicating no potential publication bias in this network meta-analysis, except for one study (32) which was outside the funnel plot.

The sensitivity analysis results showed that the estimates for treatment comparisons are very similar to those in our main analysis, despite the inclusion of the biased study. This indicates that the results of our study are robust (**Supplementary Figure 10**).

### Subgroup analysis

When analyzing HCC etiology and HBV subgroups according to the SUCRA score, NMA observed an OS benefit of HAIC-FO+sorafenib, followed by HAIC-FO and tremelimumab+Dur. The combination of cabozantinib+Ate was ranked first for PFS. In the HCV subgroup, Ate+Bev had an OS benefit over all the other treatments, followed by nivolumab, and it was ranked first based on the SUCRA score. Lenvatinib was ranked first for PFS. After stratifying by BCLC category, nivolumab and tremelimumab+Dur were ranked first in OS for BCLC B and C patients, respectively. Lenvatinib was ranked first for PFS. In MVI/PVTT/EHS and the AFP ≧400 mg/L subgroup, SUCRA scores ranked HAIC-FO+Sor as the best treatment in terms of OS and HAIC-FO as the best treatment in terms of PFS (**Table 3**).

**Table 3:** SUCRA score for OS and PFS in the entire cohort and in relative subgroups, derived from individual patient data meta-analysis.

	Entire cohort		HBV		HCV		MVI/PVTT/EHS		AFP ≥400 μg/L		BCLC B		BCLC C	
	OS	PFS	OS	PFS	OS	PFS	OS	PFS	OS	PFS	OS	PFS	OS	PFS
**HAIC-FO+sorafenib**	0.08213	0.4336	0.9882				0.9816	0.3234	0.9914					
**HAIC-FO**	0.9846	0.9816	0.8502				0.9059	0.9612	0.01276					
**Nivolumab**	0.8764	0.4715	0.4887		0.8434		0.6948	0.3396	0.8145		0.2455		0.843	
**Tremelimumab+Dur**	0.7892	0.0642	0.7383		0.3641		0.5587		0.8045		0.8085		0.7752	
**Ate+Bev**	0.6897	0.7251	0.4347	0.5385	0.9525	0.5847	0.4914	0.6054	0.4129	0.4704	0.5397	0.5759	0.752	0.6607
**Durvalumab**	0.5687	0.1666	0.443		0.2921		0.3075		0.6023		0.6624		0.3935	
**Cabozantinib+Ate**	0.5315	0.4829	0.5399	0.7975	0.02749	0.3873	0.3335	0.6198		0.04939				
**Sorafenib**	0.3825	0.5325	0.1624	0.2894	0.5395	0.2661	0.1933	0.395	0.4114	0.6416	0.4625	0.4503	0.214	0.2301
**Donatinib**	0.3273	0.7473												
**Sintilimab+BevBiol**	0.3235	0.4545	0.1494	0.2482			0.1593	0.3184	0.3765	0.5917	0.2048	0.375	0.3316	0.3934
**Lenvatinib**	0.3233	0.2897	0.2053	0.6264	0.481	0.7619	0.1561	0.1526	0.4063	0.9872	0.5767	0.5988	0.1907	0.7157
**HAIC+Sor**	0.2756	0.7718						0.7847	0.1674	0.2597				
**TACE+RT**	0.3456	0.3788												

Blank space: no data available.

HAIC-FO, hepatic arterial infusion chemotherapy-FOLFOX; HBV/HCV, hepatitis B virus (B)/hepatitis C virus (C); BCLC, Barcelona Clinic Liver Cancer; OS, overall survival; PFS, progression-free survival; TACE+RT, transarterial chemoembolization+radiotherapy; BevBiol, bevacizumab biosimilar.

### Drug safety

The included RCTs reported 15 different treatment modalities, and we compared their adverse events ≧grade 3. The network analysis result is presented in **Table 2**. Nivolumab showed a significantly lower risk of SAEs than all other treatment modalities, except for durvalumab (OR = 0.55, 95% CI = 0.36–0.84) and TACE+RT (OR = 0.62, 95% CI = 0.20–1.95), followed by durvatinib (OR = 1.11, 95% CI = 0.37–3.50), donatinib (OR = 0.83, 95% CI = 0.54–1.27), and HAIC+Sor (OR = 0.55, 95% CI = 0.29–1.06).

Heatmaps of the subgroup analyses of hematologic and non-hematologic SAEs are shown in **Figure 1**. Sorafenib caused a high percentage of neutropenia (10.7%). HAIC-Fo+sorafenib and HAIC-Cis+Sor caused a high percentage of thrombocytopenia. The top 3 non-hematological SAEs with the highest incidence were sorafenib-induced hand–foot syndrome (1.293), hypertension (1.092), and diarrhea (0.77).

## Discussion

Systemic therapies have played an important role in the treatment of aHCC for decades. The emergence of TKI therapy in 2007 and immunotherapy in 2017 paved the way for multidisciplinary therapy to gradually expand treatment options. As a result, the median OS for patients with poor prognosis in aHCC is expected to improve from 7 months to 2 years. However, for patients with aHCC complicated by PVTT, the prognosis after surgical resection is still poor. Between 44% and 62% of HCC patients will develop PVTT, and only a few who are strictly selected will undergo curative surgery. Therefore, it is necessary to identify patients who cannot undergo surgical treatment and provide more active treatment strategies to prolong their survival time (13). In recent years, with the continuous advancement of surgical technology, local therapy, radiotherapy, molecular target therapy, and immunotherapy have been combined to formulate precise treatment options to improve the prognosis of patients with aHCC complicated by PVTT.

To the best of our knowledge, this current study is the first to conduct a patient-level network analysis to comprehensively compare the benefits and safety profiles of the most updated modalities of interest for patients with aHCC-PVTT. The results of this study demonstrate that, for patients with unresectable HCC, locoregional monotherapy (HAIC-FOLFOX) or combined targeted agents (TACE+Len, HAIC+Sor, and TACE+Sor) are superior to all studied treatment modalities in terms of OS (**Table 2**). The results of this latest network analysis support the results of Deng J et al. (48) TACE+lenvatinib showed better results, but no significant advantage was found between TACE+lenvatinib and TACE+sorafenib. The results of this current network study may also be explained by the concept of the tumor microenvironment, which is mainly composed of tumor cells, infiltrating immune cells around the tumor, new vessels and endothelial cells, tumor-associated fibroblasts, and an extracellular matrix (49). The tumor microenvironment undergoes a process of dynamic change. As tumor cells proliferate indefinitely, they stimulate the production of proangiogenic factors and immunosuppressive cells, resulting in an immunosuppressive microenvironment (50–52). Lenvatinib is a novel anti-angiogenesis multikinase inhibitor, and it inhibits the combination of vascular endothelial growth factor (VEGF) and vascular endothelial growth factor receptor (VEGFR) (53). VEGF is highly expressed in HCC and is the most representative pro-angiogenic factor in the tumor microenvironment, so it is a key mediator in inhibiting the tumor microenvironment (52). Therefore, lenvatinib can alleviate immunosuppression in the tumor microenvironment by inhibiting the binding mechanism of VEGF and VEGFR, and immune checkpoint inhibitors (ICIs) work under the condition of T lymphocyte infiltration. Therefore, lenvatinib can inhibit the formation of tumor blood vessels, increase the infiltration of T lymphocytes in the immunosuppressive microenvironment, and provide an effective immunotherapy microenvironment for anti-PD-1 treatment. Therefore, lenvatinib combined with anti-PD-1 therapy has a synergistic effect (49).

Overall survival is considered the most reliable and clinically meaningful endpoint for evaluating drug efficacy in oncology trials, and it provides objective, accurate, and easy-to-interpret data in NMA studies. In our subgroup analysis, we found that age, etiology, APF, PVTT, and EHS were the main prognostic factors affecting the clinical outcome of overall survival, and this is generally consistent with the main results of the examined studies. The results of the subgroup analyses showed that, compared with all other modalities of interest, the HAIC-FO+sorafenib and HAIC-FO regimens showed significant improvements in OS in HBV and MVI/PVTT/EHS subgroups (**Table 3**). These results provide reassurance that HAIC-based target therapy may effectively control tumor burden, provide a higher response rate than MTDs or ICIs alone in patients with portal vein thrombosis or a high intrahepatic tumor burden, and reduce the risk of postoperative recurrence (1^3^).

In the SUCRA ranking plot, HAIC-FOLFOX ranked the highest in terms of OS and PFS, while HAIC-Sor ranked higher in PFS and lower in OS. The remaining MTD or ICI monotherapy rankings were comparable. The advances in molecular therapy and immune therapy are likely to have challenged the locoregional modalities with chemotherapy. However, due to the different mechanisms of both therapies, chemotherapeutic agents inhibit the DNA synthesis of the tumor, and molecular agents inhibit the multikinases involved in cell proliferation. Therefore, multikinases are limited to patients with Child–Pugh class A liver disease, and HAIC with chemotherapeutic agents benefits patients with Child–Pugh class B or C liver disease. The emergence of immune agents seems to have brought new hope to patients and oncologists regarding the systemic treatment of aHCC. Unfortunately, the development of immunotherapeutic agents appears to be limited by mechanisms involving the hyperactivation of the Wnt/β-catenin signaling pathway, which occurs in 50% of HCCs with a 5-year relapse rate of up to 70%, despite its marginal survival benefit for hepatovirus-infected HCC (54). Therefore, the replacement of conventional chemotherapy with MTA and immunotherapy is particularly controversial. Our result may accelerate studies in which MTD or ICI is added to locoregional modalities such as TACE or SBRT, as it is believed that these combinations may be sufficient to kill tumor cells and subsequently improve tumor-killing efficacy for treating aHCC (55, 56).

Another issue in this study may be heterogeneity due to the slightly different chemotherapy regimens, doses, and HAIC concomitant drug selection. We found that the dose and duration of HAIC-FOLFOX (fluorouracil, leucovorin, and oxaliplatin) used in 60% (3/5) of the locoregional studies were almost the standard starting dose and duration and that they needed to be adjusted during treatment according to the clinical condition of the patients. The doses and durations of the HAIC–cisplatin plus 5-fluorouracil and HAIC–cisplatin regimens were also similar in the remaining studies. Based on this finding, it was determined that the dose, duration, and alternative agents used in HAIC may not affect the efficacy, but special attention may need to be paid to SAEs when selecting molecular therapies or immunotherapies combined with HAIC (54).

A comparison of safety (SAE ≥ grade 3) in this NMA may be challenging, as several covariates may influence the occurrence of SAEs, such as different follow-up times and treatment durations, as well as the response of individual patients to the drugs, e.g., patient idiosyncrasy to drugs and late onset of the effects of SAEs. Therefore, simply comparing the reported rates of adverse events of any grade is not feasible to obtain a detailed comparison of the toxicity profile of the included regimens. For this reason, our analysis focused on SAE ≥grade 3 and used Bayesian NMA to optimize data extrapolation and minimize reporting bias in SAE comparisons. Regarding serious adverse events, nivolumab, durvalumab, and TACE+RT ranked in the upper left corner of the legend table due to their relatively low incidence of grade 3–5 SAEs. After integrating the SAE chromatography of the regimens of interest, we observed that nivolumab provided a lower severe toxicity than the other regimens. This result is also supported by a published study conducted by Pan et al. (55) Overall, we suggest that nivolumab might be a good alternative to sorafenib or in combination with MTD, ICI, SBRT, or TACE as a sequential line for aHCC with PVTT (13, 56, 57).

The strength of this study is that we included updated published data from phase II and III randomized clinical trials and focused on the incidence of grade III SAEs, minimizing reporting bias. The first limitation is that the reported data (PFS and OS) require a longer follow-up time; some study results may be underestimated. Second, approximately 30% of the study population consisted of 100% patients with aHCC and PVTT, and in most of the remaining studies, only more than 50% of the study population had aHCC with PVTT, so selection bias may exist. Third, given the increasing understanding of post-marketing SAE reporting analyses, the results of toxicity analyses should be interpreted with caution. Finally, the potential risks of bias may be caused by performance bias because all included randomized clinical trials employed an open-labeled study design. However, the overall assessment showed that the quality of the evidence was high. Despite these limitations, the sensitivity analysis was robust. We believe that this study can provide clinicians with new treatment options for aHCC with PVTT and improve patient survival rates and quality of life.

## Conclusion

In conclusion, PVTT remains an obstacle to the treatment of HCC, resulting in a high recurrence rate and poor prognosis. Except for sorafenib and lenvatinib, there is currently no standard treatment regimen for HCC associated with PVTT, but more active treatment modalities have been proposed and practiced clinically, which may improve the prognosis and survival time of patients with HCC associated with PVTT.

By conducting a robust NMA on individual patient-level data from RCTs, our current study provides further evidence that supports multimodality treatment as a better option for aHCC with MVI or PVTT. In view of the different efficacies observed in different subgroups (for example, HAIC-FO+sorafenib is slightly better for aHCC patients with MVI/PVTT/EHS and AFP ≧400 mg/L), multimodality treatment should be individualized by taking these subgroup factors into consideration. In the future, phase III randomized controlled trials are needed to develop better multimodality treatment strategies to manage HCC patients with PVTT.

## Data availability statement

The original contributions presented in the study are included in the article/**Supplementary Material.** Further inquiries can be directed to the corresponding authors.

## Author contributions

AC: Conceptualization, Data curation, Writing – original draft. S-YW: Writing – review & editing. JL: Software, Writing – original draft. HL: Writing – review & editing.
