# Supplementary material for: Comparative efficacy and safety of multimodality treatment for advanced hepatocellular carcinoma with portal vein tumor thrombus: patient-level network meta-analysis
Source: Front Oncol. 2024 Feb 16;14:1344798. doi: 10.3389/fonc.2024.1344798 (PMC10905023; doi:10.3389/fonc.2024.1344798)
Supplement: Supplementary file 1 [file DataSheet_1.pdf]

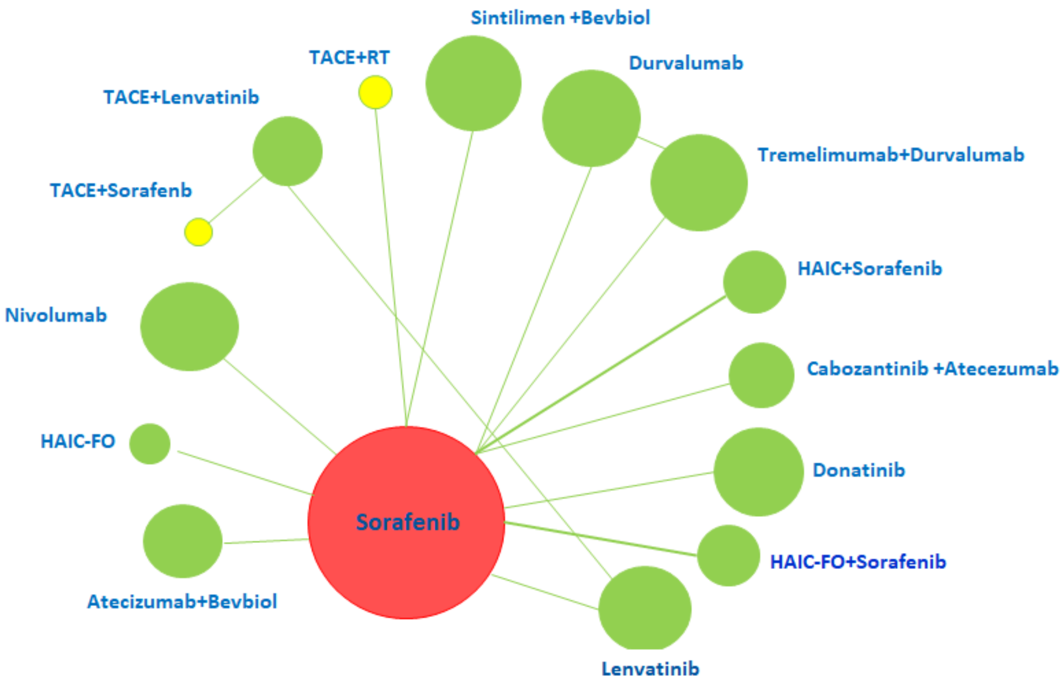

Supplementary Figure 1. Network map of randomized controlled trials comparing multimodality treatment for aHCC with PVTT

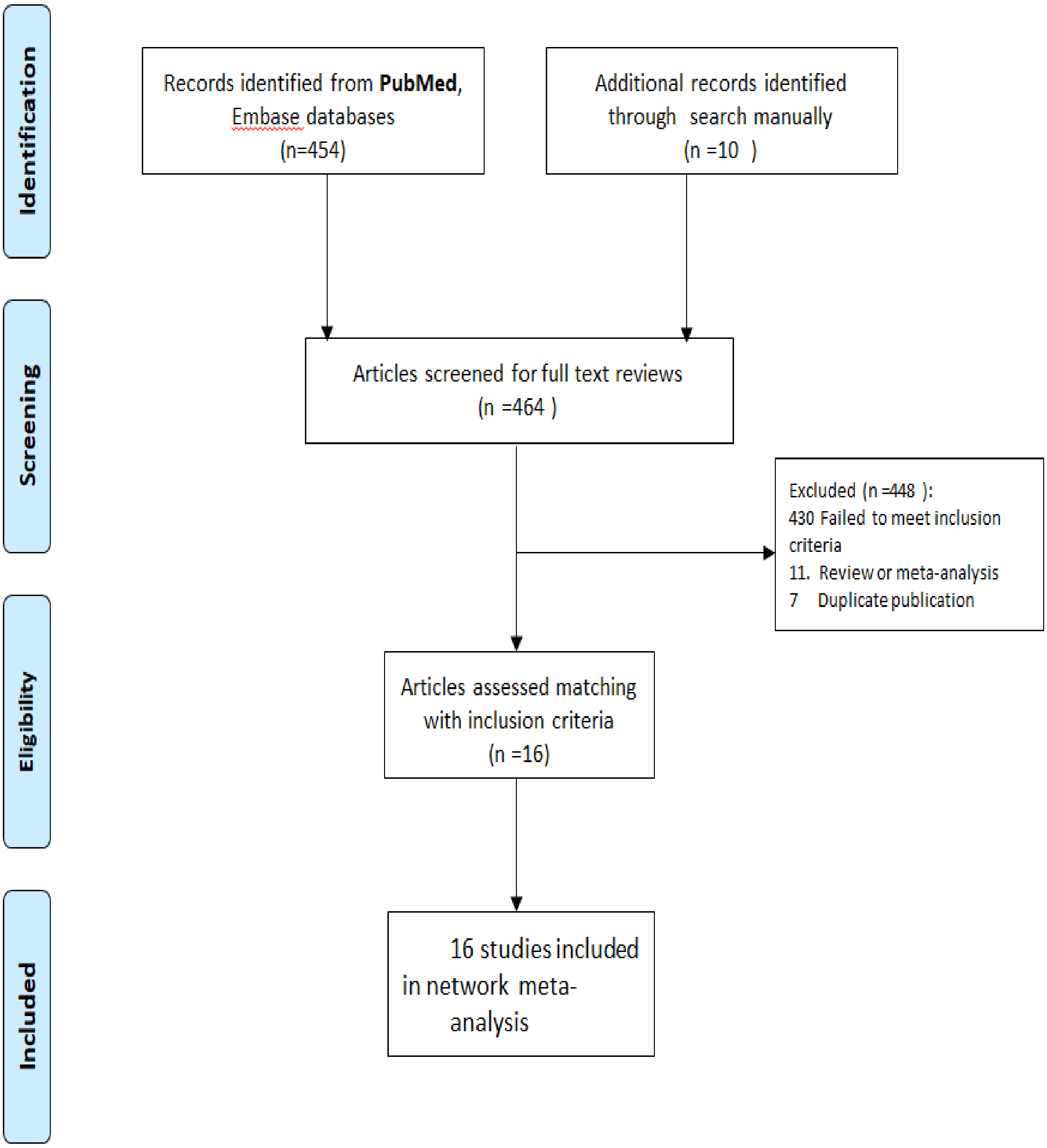

Supplementary Figure 2 The flow chart summarizing the process for the identification of the eligible clinical studies

(A)

Study or Subgroup	Odds Ratio M-H, Fixed, 95% CI
Abou-Alfa 2022	1.19 [0.75, 1.88]
Abou-Alfa Dur vs Sor	1.49 [0.98, 2.25]
Cheng, 2022	0.80 [0.47, 1.37]
Choi 2018	1.32 [0.47, 3.73]
Ding 2021	1.13 [0.42, 3.02]
He, MK, 2019	1.05 [0.63, 1.73]
Kelley 2022	1.04 [0.60, 1.81]
Kondo, 2019	0.96 [0.35, 2.62]
Kudo, 2018 Reflect	1.26 [0.88, 1.81]
Kudo 2018 Silius	0.63 [0.36, 1.10]
Lyu 2022	0.33 [0.12, 0.89]
QIN 2021	0.81 [0.60, 1.10]
Ron 2021	1.08 [0.70, 1.65]
Yau,2021	1.05 [0.76, 1.44]
Yoon 2018	1.19 [0.52, 2.73]
Zheng 2022	1.28 [0.48, 3.43]
**Total (95% CI)**	**1.02 [0.90, 1.15]**
Total events	

Heterogeneity: Chi² = 16.48, df = 15 (P = 0.35); I² = 9%
Test for overall effect: Z = 0.27 (P = 0.79)

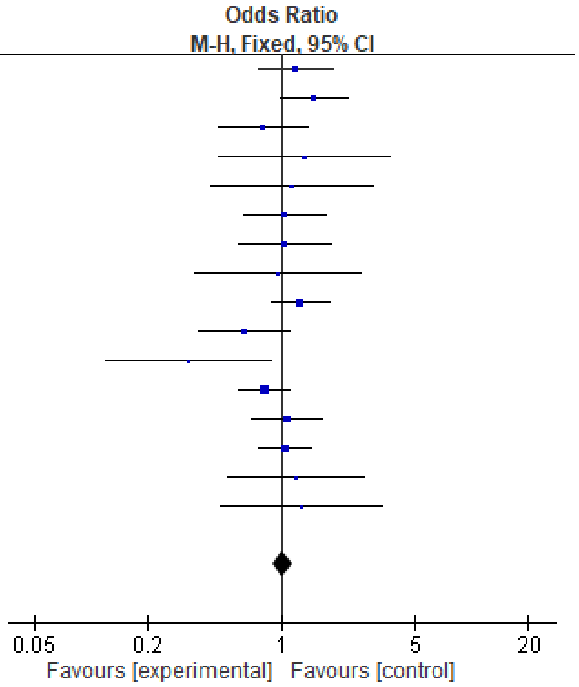

(B)

Study or Subgroup	Experimental Events	Experimental Total	Control Events	Control Total	Odds Ratio M-H, Fixed, 95% CI
Abou-Alfa 2022	262	393	280	389	0.78 [0.57, 1.06]
Abou-Alfa Dur vs Sor	280	389	293	389	0.84 [0.61, 1.16]
Cheng, 2022	146	258	77	120	0.73 [0.47, 1.14]
Choi 2018	19	29	18	29	1.16 [0.40, 3.39]
Ding 2021	16	32	15	32	1.13 [0.42, 3.02]
He, MK, 2019	78	125	63	122	1.55 [0.94, 2.58]
Kelley 2022	131	298	70	148	0.87 [0.59, 1.30]
Kondo, 2019	18	35	15	33	1.27 [0.49, 3.30]
Kudo, 2018 Reflect	259	336	250	329	1.06 [0.74, 1.52]
Kudo 2018 Silius	58	73	59	75	1.05 [0.47, 2.32]
Lyu 2022	43	70	50	59	0.29 [0.12, 0.68]
QIN 2021	115	328	112	331	1.06 [0.77, 1.46]
Ren 2021	303	380	150	191	1.08 [0.70, 1.65]
Yau,2021	186	280	206	262	0.54 [0.37, 0.79]
Yoon 2018	22	45	21	45	1.09 [0.48, 2.50]
Zheng 2022	19	32	17	32	1.29 [0.48, 3.47]
**Total (95% CI)**		**3103**		**2586**	**0.89 [0.79, 1.00]**
Total events	1955		1696		

Heterogeneity: Chi² = 24.27, df = 15 (P = 0.06); I² = 38%
Test for overall effect: Z = 2.03 (P = 0.04)

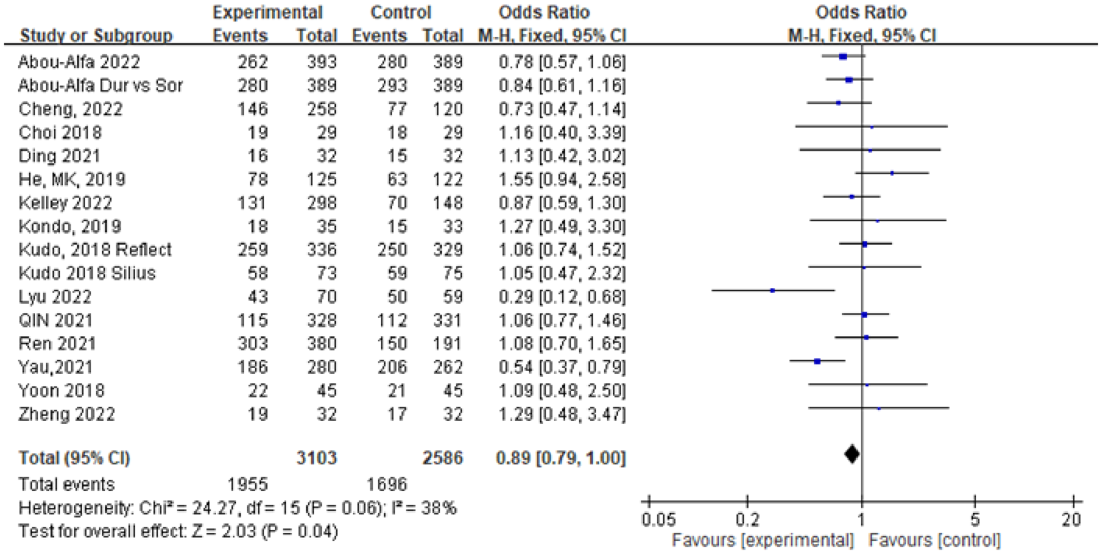

Supplementary Figure 3. I^2 test for heterogeneity in direct comparison of regimens (A) overall survival (B) progress free survival in this network analysis

A

B

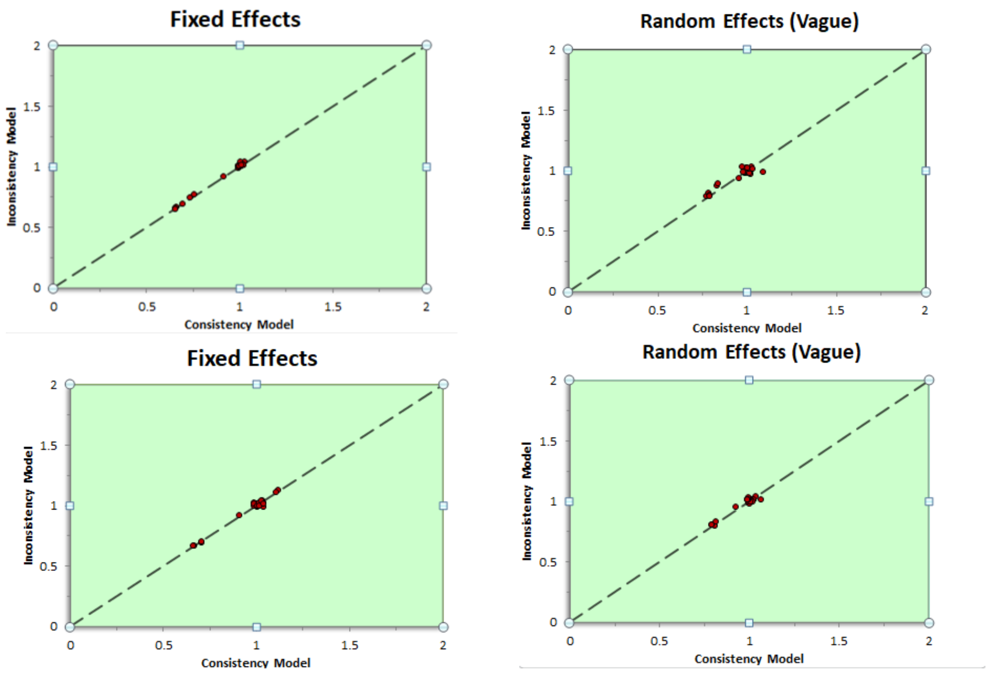

Supplementary Figure 4. Inconsistency vs consistency plot for (A) Overall survival (B) Progression free survival of multimodality treatments. Plot of individual data points for the consistency model (horizonal axis) and the inconsistency model (vertical axis), along with the equality line.

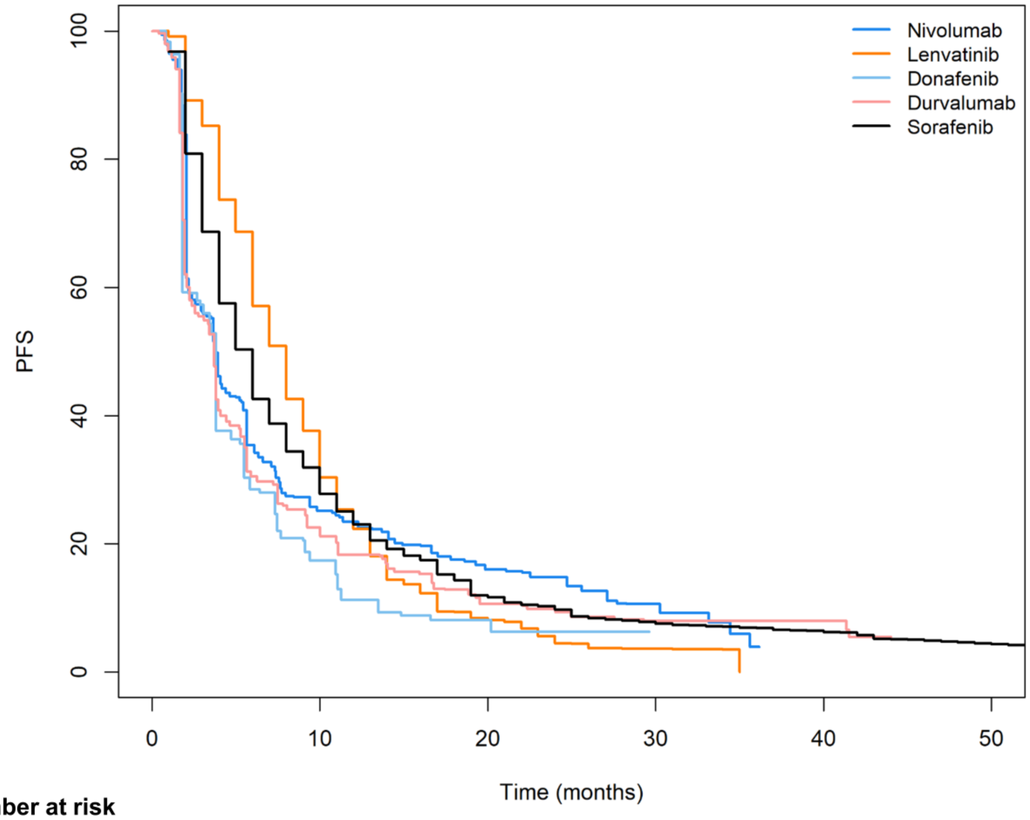

Number at risk

	0	10	20	30	40	50
Nivolumab	371	84	40	11	0	0
Lenvatinib	478	158	29	4	0	0
Donafenib	328	29	7	0	0	0
Durvalumab	393	91	46	26	1	0
Sorafenib	2699	946	409	201	122	47

Supplementary Figure 5. Kaplan-Meier curves for PFS for monotherapy regimens

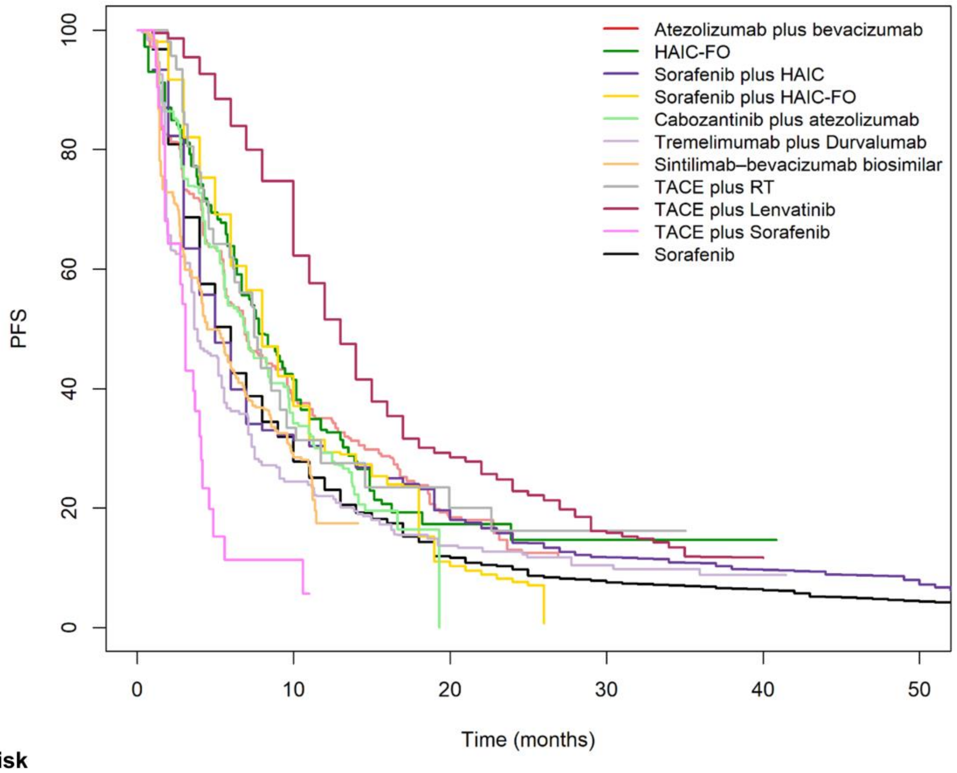

Number at risk

Atezolizumab plus bevacizumab	165	113	41	0	0	0
HAIC-FO	130	49	17	6	1	0
Sorafenib plus HAIC	150	81	44	22	10	2
Sorafenib plus HAIC-FO	157	49	22	0	0	0
Cabozantinib plus atezolizumab	250	66	0	0	0	0
Tremelimumab plus Durvalumab	389	88	38	20	5	0
Sintilimab–bevacizumab biosimilar	380	37	0	0	0	0
TACE plus RT	45	27	5	1	0	0
TACE plus Lenvatinib	202	137	42	11	0	0
TACE plus Sorafenib	32	2	0	0	0	0
Sorafenib	2699	946	409	201	122	47

Supplementary Figure 6. Kaplan-Meier curves for PFS for combination regimens

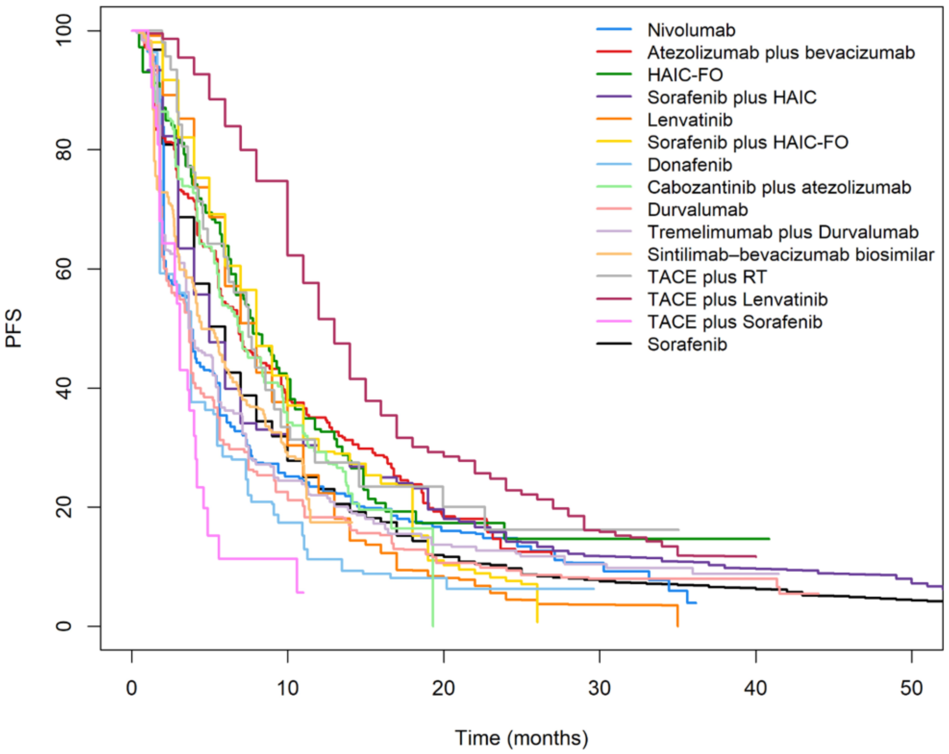

Number at risk

Nivolumab	371	84	40	11	0	0
Atezolizumab plus bevacizumab	165	113	41	0	0	0
HAIC-FO	130	49	17	6	1	0
Sorafenib plus HAIC	150	81	44	22	10	2
Lenvatinib	478	158	29	4	0	0
Sorafenib plus HAIC-FO	157	49	22	0	0	0
Donafenib	328	29	7	0	0	0
Cabozantinib plus atezolizumab	250	66	0	0	0	0
Durvalumab	393	91	46	26	1	0
Tremelimumab plus Durvalumab	389	88	38	20	5	0
Sintilimab–bevacizumab biosimilar	380	37	0	0	0	0
TACE plus RT	45	27	5	1	0	0
TACE plus Lenvatinib	202	137	42	11	0	0
TACE plus Sorafenib	32	2	0	0	0	0
Sorafenib	2699	946	409	201	122	47

Supplementary Figure 7. Kaplan-Meier curves for progression-free survival for all multimodality regimens

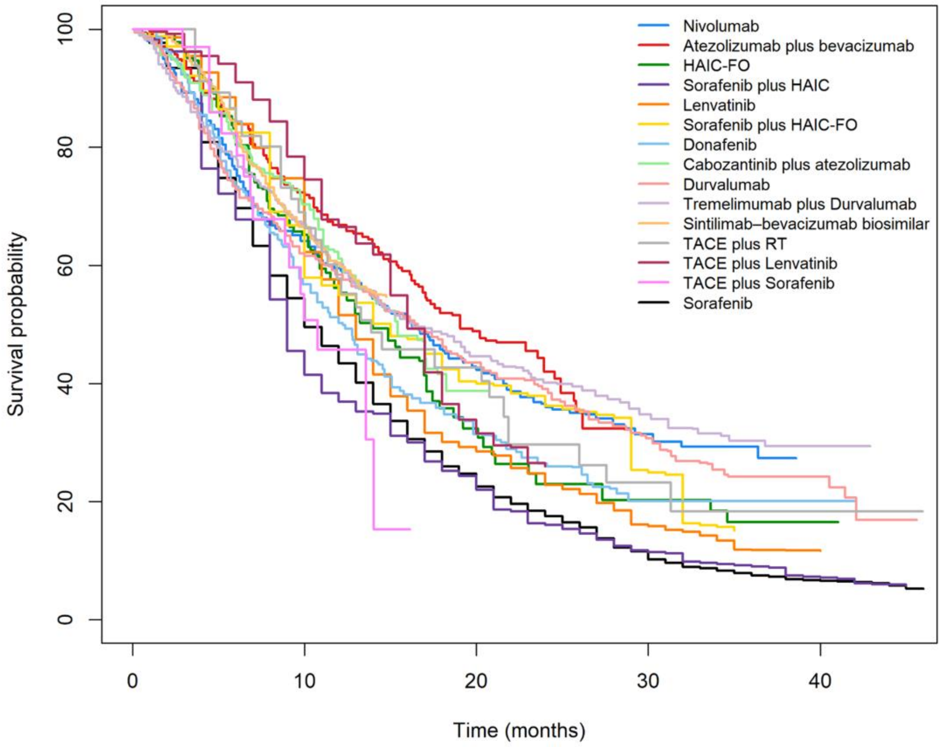

Number at risk

Nivolumab	371	216	147	63	0
Atezolizumab plus bevacizumab	336	233	134	0	0
HAIC-FO	130	76	31	10	0
Sorafenib plus HAIC	166	87	48	22	3
Lenvatinib	646	443	138	41	0
Sorafenib plus HAIC-FO	157	77	28	3	0
Donafenib	328	188	95	25	2
Cabozantinib plus atezolizumab	432	208	0	0	0
Durvalumab	389	256	161	87	8
Tremelimumab plus Durvalumab	389	71	35	20	1
Sintilimab–bevacizumab biosimilar	380	126	0	0	0
TACE plus RT	45	30	18	6	2
TACE plus Lenvatinib	202	121	23	0	0
TACE plus Sorafenib	32	12	0	0	0
Sorafenib	2637	1339	446	66	0

Supplementary Figure 8. Kaplan-Meier curves for overall survival for all multimodality regimens

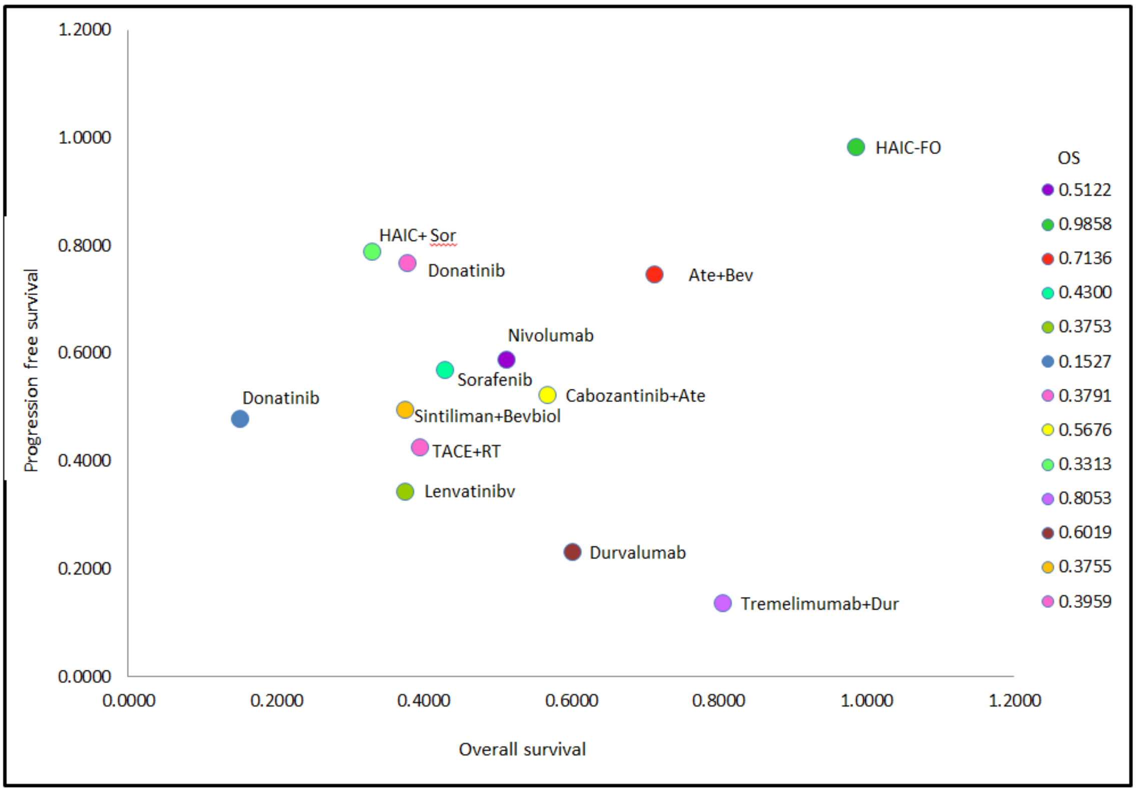

Supplementary Figure 9. SUCRA ranking probabilities of regimens for PFS and OS. Abbreviations: HAIC (hepatic arterial infusion chemotherapy

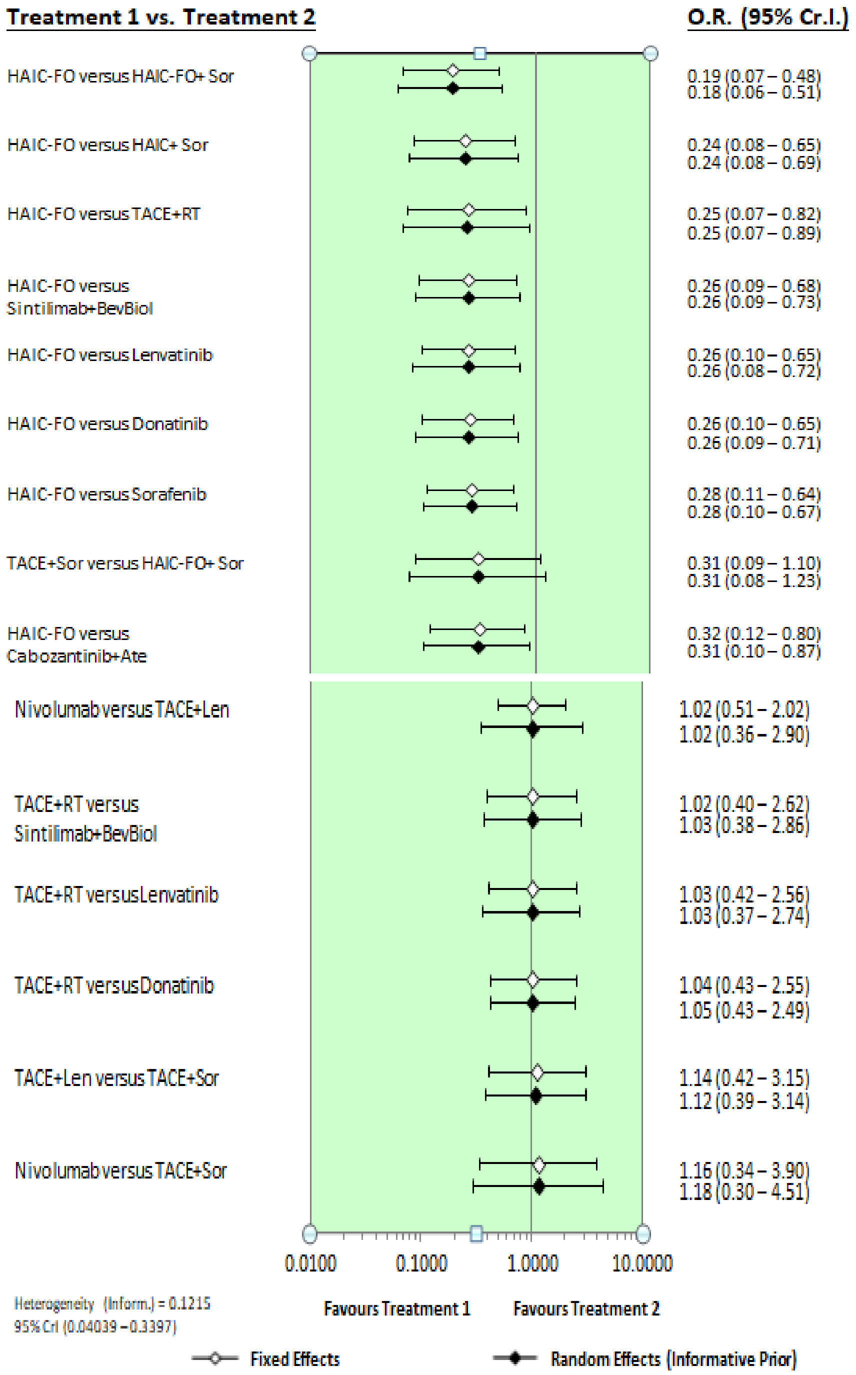

Treatment 1 vs. Treatment 2
O.R. (95% Cr.I.)
HAIC-FO versus HAIC-FO+ Sor
0.19 (0.07 – 0.48)
0.18 (0.06 – 0.51)
HAIC-FO versus HAIC+ Sor
0.24 (0.08 – 0.65)
0.24 (0.08 – 0.69)
HAIC-FO versus TACE+RT
0.25 (0.07 – 0.82)
0.25 (0.07 – 0.89)
HAIC-FO versus Sintilimab+BevBiol
0.26 (0.09 – 0.68)
0.26 (0.09 – 0.73)
HAIC-FO versus Lenvatinib
0.26 (0.10 – 0.65)
0.26 (0.08 – 0.72)
HAIC-FO versus Donatinib
0.26 (0.10 – 0.65)
0.26 (0.09 – 0.71)
HAIC-FO versus Sorafenib
0.28 (0.11 – 0.64)
0.28 (0.10 – 0.67)
TACE+Sor versus HAIC-FO+ Sor
0.31 (0.09 – 1.10)
0.31 (0.08 – 1.23)
HAIC-FO versus Cabozantinib+Ate
0.32 (0.12 – 0.80)
0.31 (0.10 – 0.87)
Nivolumab versus TACE+Len
1.02 (0.51 – 2.02)
1.02 (0.36 – 2.90)
TACE+RT versus Sintilimab+BevBiol
1.02 (0.40 – 2.62)
1.03 (0.38 – 2.86)
TACE+RT versus Lenvatinib
1.03 (0.42 – 2.56)
1.03 (0.37 – 2.74)
TACE+RT versus Donatinib
1.04 (0.43 – 2.55)
1.05 (0.43 – 2.49)
TACE+Len versus TACE+Sor
1.14 (0.42 – 3.15)
1.12 (0.39 – 3.14)
Nivolumab versus TACE+Sor
1.16 (0.34 – 3.90)
1.18 (0.30 – 4.51)
0.0100
0.1000
1.0000
10.0000
Heterogeneity (Inform.) = 0.1215
95% CrI (0.04039 – 0.3397)
Favours Treatment 1
Favours Treatment 2
Fixed Effects
Random Effects (Informative Prior)

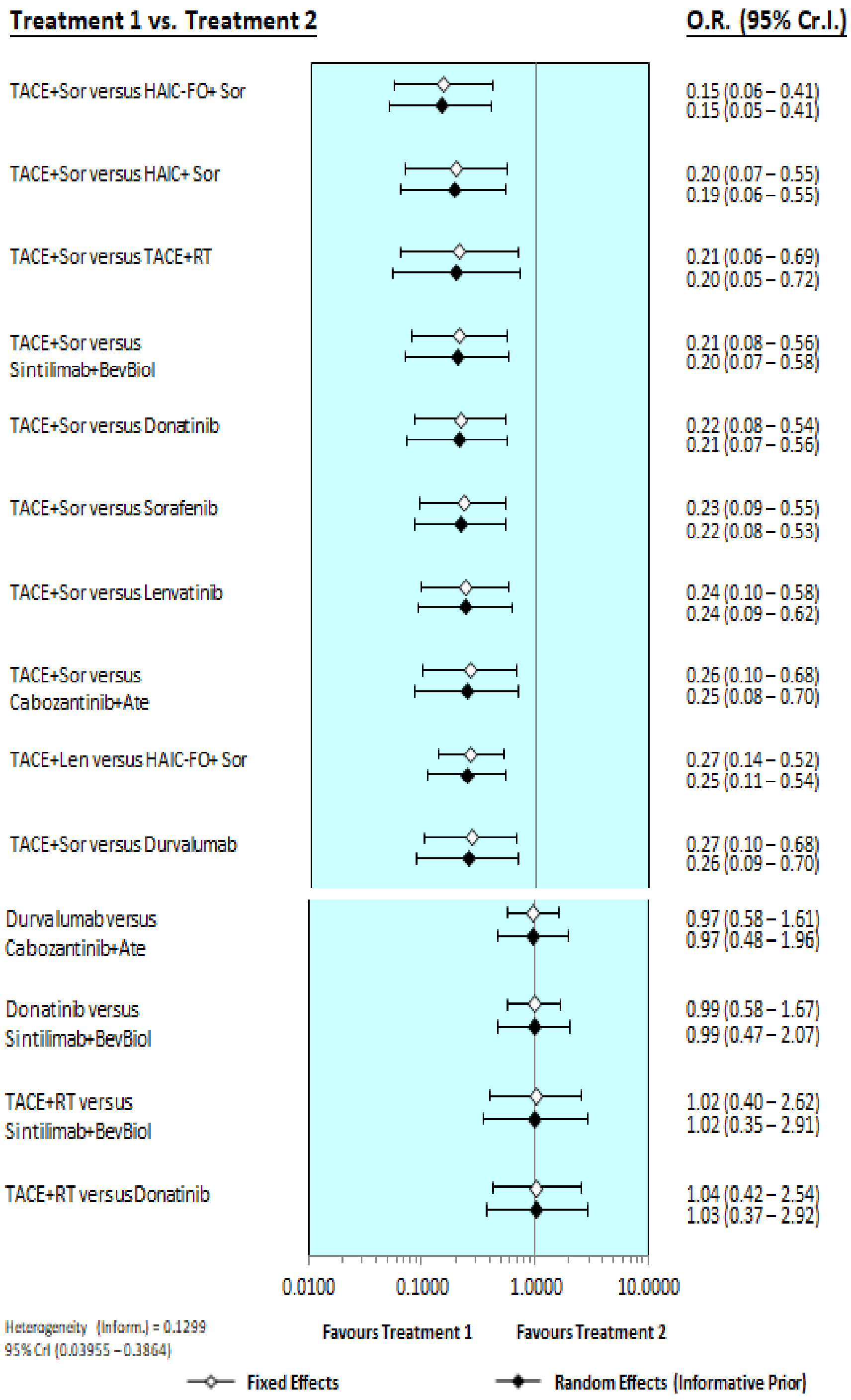

Supplementary Figure 10.Sensitivity analysis results in terms of forest plot including a study (A) without high risk bias, (B) with high risk bias. Both forest plot exhibited similar OR value and heterogeneity.
